# Scattering properties of Intralipid modeled using dependent scattering theory

**DOI:** 10.1117/1.JBO.31.8.085003

**Published:** 2026-08-27

**Authors:** Xavier Attendu, Martin Poinsinet de Sivry-Houle, Mona Shahsavari, Dirk J. Faber, Edwin van der Pol, Ton G. van Leeuwen

**Affiliations:** a Amsterdam UMC, Location University of Amsterdam, Department of Biomedical Engineering and Physics, Amsterdam, The Netherlands; b Scinvivo B.V., Eindhoven, The Netherlands

**Keywords:** biophotonics, dependent scattering theory, optical phantoms, intralipid

## Abstract

**Significance:**

Intralipid^®^ is widely used as a liquid optical phantom in biomedical optics, but its scattering properties remain incompletely defined, particularly at high volume fractions where dependent scattering becomes important. A physics-based description of its scattering behavior is needed to support quantitative Monte Carlo simulations, phantom design, and calibration of biophotonic measurement systems.

**Aim:**

We aim to establish a physically grounded model of Intralipid scattering, including the scattering coefficient, reduced scattering coefficient, anisotropy, and phase function. The model covers wavelengths 400 to 1700 nm and particle volume fractions of 0.00227 to 0.227, corresponding to dilutions from 1% v/v of stock Intralipid 20% through undiluted stock Intralipid 20%. It is derived from intrinsic material properties, namely, the particle size distribution and the refractive indices of the lipid particles and aqueous medium.

**Approach:**

We combine full Mie theory for the single-particle scattering response with the multicomponent Percus–Yevick hard-sphere approximation to account for dependent scattering. The model uses recent measurements of the particle size distribution and the refractive-index dispersion of the lipid and aqueous phases. Particular attention is given to the long tail of the particle size distribution, which extends beyond the cutoff of earlier electron-microscopy-based datasets.

**Results:**

Despite its small number fraction, the long tail of the particle size distribution contributes substantially to the scattering coefficient, anisotropy, and phase function, especially at near-infrared wavelengths. The resulting predictions for the scattering coefficient, anisotropy, and reduced scattering coefficient are broadly consistent with established semi-empirical relations for Intralipid in the dependent-scattering regime, although residual wavelength- and concentration-dependent discrepancies remain. The model also provides phase functions across the full studied wavelength and concentration range, for which direct experimental reference data are currently unavailable.

**Conclusion:**

The proposed model links the optical scattering properties of Intralipid to measured material properties while incorporating dependent-scattering effects. Its broad agreement with literature-reported data on IL optical properties supports its use as a physics-based estimate, while the remaining discrepancies highlight the influence of sample variability and the limits of the underlying assumptions. The reported equations and interpolation scripts provide a practical resource for studies requiring internally consistent scattering properties across wavelength and concentration.

## 1 Introduction

Intralipid^®^ (IL) is a lipid emulsion widely utilized as a liquid, tissue-mimicking optical phantom due to its low cost, stability, and reproducible optical properties (specifically scattering coefficient $\mu_{s}$, reduced scattering coefficient $\mu_{s}^{\prime }$, anisotropy g, and phase function p(θ)).^1^^–^^3^ These phantoms are essential for calibrating biophotonic modalities such as diffuse reflectance spectroscopy,^4^ single fiber reflectance spectroscopy,^5^ or optical coherence tomography.^6^ However, the reliability of these calibrations is constrained by the degree of accuracy with which the optical properties are known. Although extensive literature exists characterizing these properties,^3^^,^^7^ experimental quantification remains inconsistent. Even with robust, multimodal measurement strategies, systematic uncertainties related to calibration standards, model inversions, and detector geometries prevent the emergence of a consensus dataset. Critically, there is at present no widely adopted physics-based reference to help interpret and reconcile these conflicting macroscopic measurements. This absence is most acute for the scattering phase function, which is notoriously difficult to measure experimentally yet is a requisite input for the high-fidelity Monte Carlo simulations that underpin many modern device validation workflows.

Establishing a theoretical benchmark is complicated by the gradual transition from independent scattering (IS) for highly dilute samples to dependent scattering (DS) for IL solutions with low dilution factors (i.e., high scatterer volume fraction). In the IS regime, the positions of scatterers are statistically uncorrelated, and bulk properties can be computed using Mie theory and an incoherent summation of scattered intensities (i.e., interference effects are neglected). However, measurable deviations from IS predictions have been reported at volume fractions, $\phi_{p}$, as low as 2% to 3%.^3^^,^^8^ With increasing particle volume fraction, steric exclusions progressively impose short-range order on the particle distribution, inducing spatial correlations and coherent interference effects between the scattered fields that increasingly alter the bulk scattering properties. Under IS, the scattering coefficient scales linearly with particle volume fraction, while the phase function and anisotropy are concentration independent. Concentrated IL does not follow these predictions (as illustrated in Fig. 7). For example, at λ=800  nm and $\phi_{p}=0.114$, IS overestimates $\mu_{s}$ by more than 40% compared with both experimental data^3^^,^^9^ and the presented DS model. Consequently, IS alone cannot adequately predict optical properties for concentrated samples, and accurate modeling of concentrated IL samples requires a rigorous treatment of many-body interactions.

To address this, the multicomponent Percus–Yevick hard-sphere approximation (PYHSA) has been proposed as a method to model dependent scattering in polydisperse suspensions. However, previous attempts to apply this framework to Intralipid^10^^,^^11^ have been fundamentally limited by the quality of available input parameters. Historically, modeling efforts have relied on a single particle size distribution (PSD) measured via electron microscopy in 1991.^2^ Although foundational, this dataset suffered from coarse binning (50 nm width), a low size cutoff (max. particle diameter of 675 nm), and low particle counts, introducing potential statistical uncertainties affecting dependent scattering calculations. Furthermore, these models have often simplified the refractive index of the lipid phase, assuming a constant refractive index offset relative to water rather than treating the specific dispersion relation of soybean oil. Finally, these works rarely provided the resulting phase functions in an accessible format, limiting their practical utility for calibrating modern biophotonic instrumentation.

In this work, we modernize the application of the multicomponent PYHSA to generate a physics-based benchmark for Intralipid. We leverage high-precision measurements recently reported by our group^12^ regarding the PSD and refractive index curves for soybean oil to constrain the model with superior input data. This approach allows us to: (1) generate physics-based predictions for $\mu_{s}$, $\mu_{s}^{\prime }$, and g across the full concentration range ($0.00227<\phi_{p}\leq 0.227$), demonstrating broad consistency with established experimental trends; (2) predict the complete scattering phase function p(θ), providing a reference for regimes where macroscopic measurements are inaccessible^13^; and (3) provide the community with explicit equations and lookup tables to compute these properties across the studied volume-fraction range and the biologically relevant VIS–NIR wavelength range (400 to 1700 nm).

## 2 Theory & Methods

### 2.1 Theoretical Framework & Model Assumptions

#### 2.1.1 Particle properties

The PYHSA treats IL as a polydisperse suspension of hard, homogeneous dielectric spheres (soybean oil) in a medium (water), with negligible long-range electrostatic interactions. The spherical shape is supported by transmission electron microscopy (TEM) measurements by van Staveren et al., who also validated that small deviations from the ideal spherical shape did not significantly impact the Mie scattering calculations.^2^ Moreover, it is known that IL particles consist of a phospholipid monolayer (egg lecithin emulsifier) surrounding the lipid core.^7^ This outer shell was not considered in Mie calculations because of its very small thickness (2 to 5 nm) compared with the total particle size as well as the expected low index contrast with the soybean oil.^2^ This approximation was further assessed by explicitly comparing homogeneous sphere and core-shell Mie models while sweeping the shell refractive index over the interval 1.457 to 1.497 (±0.01 around the lipid core value of 1.477) and assuming a 5 nm shell thickness; over particle diameters of 57 to 2000 nm and wavelengths of 400 to 1700 nm, the resulting scattering cross sections differed by <5% in the worst case (λ=400  nm and 57 nm diameter) and <1% for the most abundant particles (∼400  nm), indicating weak sensitivity to the shell parameters despite their uncertainty. The imaginary component of the refractive index (κ) was not used in the simulations as it was found to have a negligible impact (<0.01% relative change based on data from Ref. 14) on the computed scattering properties. Finally, the lipid particles possess a relatively strong electrostatic potential (negative ζ-potential of approximately 40 to 50 mV),^15^ which confers the colloidal dispersion high stability and prevents particle aggregation. These properties support the assumption of a stable PSD invariant with dilution^7^ but would tend to contradict the noninteracting assumption made by the PYHS model. However, in the electrolytic medium, the electrostatic interaction distance described by the Debye length is expected to be ∼5  nm (approximated using an electrical conductivity of 0.04 S/m^16^ following the approximate conversion presented in chapter 2 of Ref. 17). As such, because the Debye length is far smaller than the particle size, we expect electrostatic effects to be minor, supporting the use of the noninteracting, hard-sphere approximation.

#### 2.1.2 Electromagnetic scattering

In this paper, we utilize a first-order, far-field solution of the Foldy-Lax equation, in which the excitation field at each particle is approximated by the coherent incident plane wave. The single-particle response itself is computed using full Mie theory. Dependent scattering is then included through the ensemble-averaged interference of the singly scattered far fields, while higher-order electromagnetic coupling between particles is neglected. As a result, we omit two effects: (i) recurrent radiative re-excitation by fields scattered from neighboring particles and (ii) near-field (evanescent) inter-particle coupling. These simplifications are physically motivated for IL because the lipid droplets in water are weak scatterers due to the low refractive-index contrast, $|n_{\mathrm{rel}}-1|=|n_{\mathrm{lipid}}/n_{\mathrm{water}}-1|\approx 0.1$. Under this condition, the field scattered by a droplet is only a small perturbation to the local field, so radiative re-excitation contributes only a minor correction to the local excitation field.^18^ Likewise, the associated evanescent near fields are weak and decay rapidly with separation, so near-field coupling is assumed to be a negligible contribution.

#### 2.1.3 Static structure factor

In light of the approximations presented in the paragraph above, we model the total scattered field as a superposition of single-scattered waves by each of the particles in the suspension. As we depart from the independent scattering regime (i.e., for increasing volume fraction), a certain short-range order appears in the particle positions leading to nonnegligible interference effects. In polydisperse suspensions, this short-range order is captured by a grid of pairwise total correlation functions $h_{\alpha \beta }(r)$, one for each pair of particle diameters α and β, where r is the distance between particles. In turn, the interference effect is described by a grid of $H_{\alpha \beta }(q=2k\;\sin\;(\theta /2))$ functions defined as the Fourier transforms of the corresponding $h_{\alpha \beta }(r)$ functions. Here, q is the scattering vector defined by the wavenumber k and the angle θ between the incident and scattered waves. In this work, the $H_{\alpha \beta }$ functions used are the solutions to the Ornstein–Zernike equation under the PYHSA, as derived by Baxter et al.^19^ Specifically, we used the methods outlined in chapter 8 of Ref. 20. These solutions have been extensively validated via Monte Carlo simulations^21^^,^^22^ and experimental measurements.^23^^,^^24^ Relevant derivations and MATLAB scripts are available in the appendix and online in the repository (https://doi.org/10.5281/zenodo.20207810).

### 2.2 Computation of Optical Properties

In this section, we present the equations obtained from Fujii et al.,^11^ used to calculate the optical properties of interest, namely, the scattering coefficient, $\mu_{s}$, the normalized scattering phase function, p(θ), the scattering anisotropy, g, and the reduced scattering coefficient, $\mu_{s}^{\prime }$. Assuming an incident wave propagating in the z-direction and polarized in the x-direction, the Mie scattering amplitude vector, ${\vec{F}}_{\mathrm{Mie}}^{\alpha }(\theta ,\phi )$ of each individual particle of size $d_{\alpha }$ is given by:

$${\overset{\to }{F}}_{\mathrm{Mie}}^{\alpha }(\theta ,\phi )=\frac{i}{k}[{\overset{\to }{e}}_{\theta }S_{2}(\theta ;d_{\alpha })\cos\;\phi -{\overset{\to }{e}}_{\phi }S_{1}(\theta ;d_{\alpha })\sin\;\phi ],$$ (1)

where i is the imaginary unit, k is the wavenumber of the incident wave in the medium, θ and ϕ are the polar and azimuthal scattering angles, respectively, and ${\vec{e}}_{\theta ,\phi }$ are the corresponding unit vectors in the spherical-coordinate system. $S_{1,2}(\theta ,d_{\alpha })$ are the Mie amplitude functions for a particle of diameter $d_{\alpha }$.^25^ Practically, Mie solutions were obtained through the Mie solver MatScat.^26^ The macroscopic differential scattering coefficient in all directions is defined as:

$$\frac{d\mu_{s}}{d\Omega }(\theta ,\phi )=\underset{\mathrm{independent}\;\mathrm{scattering}}{\underset{⏟}{\underset{\alpha }{\sum }n_{\alpha }{\left|{\overset{\to }{F}}_{\mathrm{Mie}}^{\alpha }(\theta ,\phi )\right|}^{2}}}+\underset{\mathrm{dependent}\;\mathrm{scattering}}{\underset{⏟}{\underset{\alpha }{\sum }\underset{\beta }{\sum }\sqrt{n_{\alpha }n_{\beta }}{\overset{\to }{F}}_{\mathrm{Mie}}^{\alpha }(\theta ,\phi )\cdot {\overset{\to }{F}}_{\mathrm{Mie}}^{\beta *}(\theta ,\phi )H_{\alpha \beta }(\theta )}},$$ (2)

where $n_{\alpha }$ and $n_{\beta }$ are the number densities of the particle size classes α and β, respectively, and $H_{\alpha \beta }(\theta )$ is the Fourier transform of the total correlation function for the pair of particle size classes with diameters $d_{\alpha }$ and $d_{\beta }$, as derived by Baxter^19^ (see Appendix 7.1 for complete equations to compute $H_{\alpha \beta }$). Integration of Eq. (2) over the unit sphere, denoted by $\int_{S^{2}}d\Omega$, with dΩ=sin θdθdϕ such that $\int_{S^{2}}d\Omega =\int_{0}^{2\;\pi }\int_{0}^{\pi }\;\sin\;\theta d\theta d\phi$, yields the scattering coefficient, $\mu_{s}$:

$$\mu_{s}=\underset{\mathrm{independent}\;\mathrm{scattering}}{\underset{⏟}{\underset{\alpha }{\sum }n_{\alpha }\sigma_{s,\mathrm{Mie}}^{\alpha }}}+\underset{\mathrm{dependent}\;\mathrm{scattering}}{\underset{⏟}{\int_{S^{2}}d\Omega \underset{\alpha }{\sum }\underset{\beta }{\sum }\sqrt{n_{\alpha }n_{\beta }}{\overset{\to }{F}}_{\mathrm{Mie}}^{\alpha }(\theta ,\phi )\cdot {\overset{\to }{F}}_{\mathrm{Mie}}^{\beta *}(\theta ,\phi )H_{\alpha \beta }(\theta )}},$$ (3)

where $\sigma_{s,\mathrm{Mie}}^{\alpha }$ is the scattering cross-section for a particle with diameter $d_{\alpha }$, equal to the integral of the magnitude squared of the Mie scattering amplitude vector [Eq. (1)] over the unit sphere. From Eq. (2), we can also obtain the normalized phase function p(θ) by averaging over all ϕ and normalizing such that $\int_{S^{2}}d\Omega p(\theta )=1$:

$$p(\theta )=\frac{1}{\mu_{s}}\cdot \frac{1}{2\pi }\int_{0}^{2\;\pi }d\phi \frac{d\mu_{s}}{d\Omega }(\theta ,\phi ).$$ (4)

From the normalized phase function, we can then compute the anisotropy g as:

$$g=2\pi \int_{0}^{\pi }d\theta \;\cos\;\theta \cdot p(\theta )\cdot \;\sin\;\theta .$$ (5)

Finally, the reduced scattering coefficient is computed as:

$$\mu_{s}^{\prime }=\mu_{s}\cdot (1-g).$$ (6)

### 2.3 Simulation Input Parameters

#### 2.3.1 Particle size distribution

**Fig. 1:**
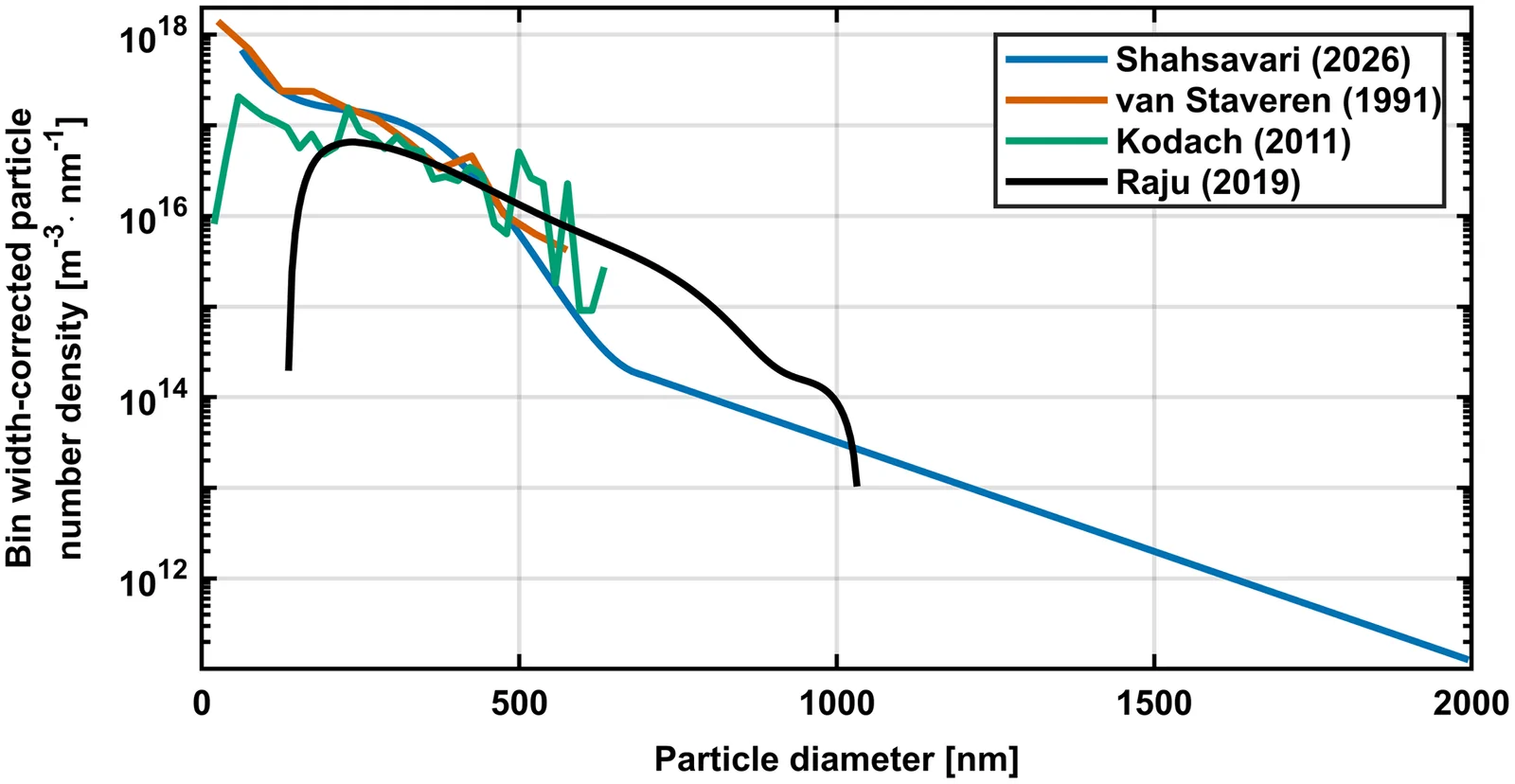
Comparison of reported particle size distributions for stock intralipid 20%. PSD data for Shahsavari et al. are obtained from the empirical Eq. (26), derived from 171 size bins with centers spanning from 57 to 2078 nm.^12^ PSD data for van Staveren et al. includes 13 size bins with centers spanning 25 to 675 nm.^2^ PSD data for Kodach et al. includes 36 size bins with centers spanning 19.2 to 691.2 nm.^27^ PSD data for Raju and Unni include 18 size bins with centers spanning 134.2 to 1035.0 nm.^28^.

A literature search yielded four different datasets providing the distinct particle size distributions for IL. These PSDs are reported in Fig. 1. To account for the variable binning of particle size, the distributions were converted to number density per nm (i.e., number density divided by bin width) and scaled to achieve a total volume fraction of 0.227, corresponding to stock IL20%.^3^ Of these studies, two determined the PSD with electron microscopy (EM),^2^^,^^27^ one used dynamic light scattering (DLS),^28^ and, most recently, our group utilized flow cytometry (FCM)^12^ leveraging an advanced calibration method for absolute size quantification.^29^ The PSD measured by van Staveren et al.^2^ was for IL10% stock from Kabivitrum (Sweden), while Refs. 12, 27, and 28 measured IL20% stock from Fresenius Kabi (Netherlands). Figure 1 illustrates that the PSDs from Refs. 2, 12, and 27 are in relatively good agreement, while Ref. 28 differs significantly. This is likely due to the use of dynamic light scattering to measure the PSD, as DLS is known to generate inaccuracies when applied to highly polydisperse samples.^30^ Other sources of variability include batch variability, minor differences based on formulation (depending on manufacturer and production year), and statistical uncertainty, particularly in the EM-based measurements. Stock concentration (i.e., 10% versus 20%) is not expected to have a major influence on the PSD.

Beyond the differences in the overlap range, it is apparent that the PSDs based on EM and DLS do not include the so-called long tail or particles in the range of 600 to 2000 nm. Its omission in EM-based PSDs is likely a consequence of limited particle counts, whereas our FCM measurement of more than 10 million particles provides the statistical sensitivity needed to detect and quantify this low-abundance tail.^12^ Moreover, the existence of larger particles in IL is an established fact^31^ and is even the object of the PFAT(5) pharmacopeial standard, limiting the volume-weighted fraction of particles exceeding 5  μm in diameter to <0.05%.^32^

For the simulations presented in this work, we therefore adopt the FCM-derived PSD from Ref. 12 as the baseline input, as it provides the broadest size coverage and the most robust counting statistics while being directly calibrated for absolute size. The remaining PSDs are included in Fig. 1 to summarize the range of literature reports and to provide context for expected variability but are not used as primary simulation inputs.

#### 2.3.2 Dispersion curves

**Fig. 2:**
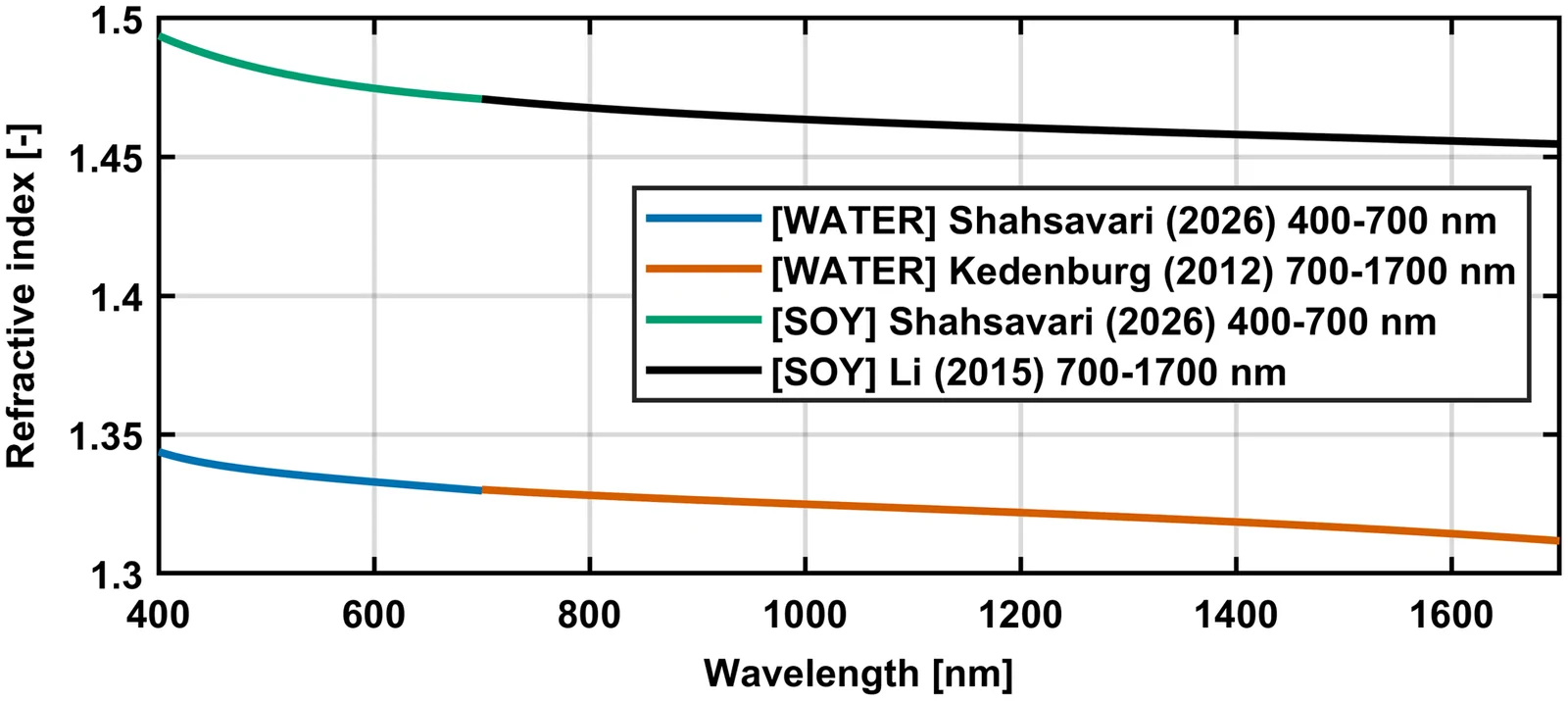
Dispersion curves for water and soybean oil across 400 to 1700 nm obtained by merging datasets available in the literature.^12^^,^^14^^,^^33^.

The dispersion curves used for the simulations presented in this manuscript are plotted in Fig. 2. For water (medium), we utilize the measurements from Ref. 12 for the specified range of 400 to 700 nm, and the reference curve from Kedenburg et al.^33^ for 700 to 1700 nm. The dispersion curve by Kedenburg et al. can also be extended to cover 400 to 700 nm and is in excellent agreement with data from Shahsavari et al. (RMS absolute difference $<5\times 10^{-4}$).^12^

Data for the refractive index of soybean oil is much more sparse. Most investigations^3^^,^^11^^,^^28^ into the optical properties of IL refer back to van Staveren et al.^2^ and define the refractive index of soybean oil as the curve for water offset by a constant: $n_{\mathrm{soy}}(\lambda )=n_{H_{2}O}(\lambda )+0.14$. More recently, our group measured the refractive index curve for soybean oil between 400 and 700 nm with metrological traceability (absolute uncertainties $<1.4\times 10^{-4}$ in all instances).^12^ For the spectral range of 700 to 1700 nm, we used data from Li et al.^14^ adjusted upwards by $7\times 10^{-4}$ (well within their reported error of ∼0.01) to match the endpoint of our data at 700 nm. Precise coefficients for all dispersion curves used in simulations can be found in Appendix 7.2.

### 2.4 Simulation Parameter Sensitivity Check

To ensure the accuracy and robustness of the dependent scattering calculations, two critical numerical approximations were evaluated: the discretization of particle size bins and the upper truncation limit of the PSD. First, we addressed the sensitivity of the simulated optical properties to bin width. Because the number density derived for a fixed volume fraction scales inversely with the cube of the diameter ($N\propto d^{-3}$), coarse binning introduces asymmetric errors in the estimation of inter-particle spacing. For example, within a bin spanning 40 to 60 nm, treating 40 nm particles as 50 nm spheres underestimates the local population density by ∼50%, artificially inflating the average inter-particle separation and altering the calculated interference effects. Sampling density is also relevant for the computation of dependent scattering effects as the number of total correlation functions [$H_{\alpha \beta }(\theta )$ in Eq. (3)] to compute scales with the square of the number of size bins. To assess the effect of binning, we implemented both linear and logarithmic binning at multiple different sampling resolutions over the size range 50 to 2000 nm. These analyses were carried out on the PSD measured by FCM, where fine resampling was possible using Eq. (26).^12^ The method for PSD definition with different binning resolutions is presented in Appendix 7.2.

Second, the PSD measured by FCM extends significantly beyond the historical limit of 675 nm utilized in van Staveren’s dataset.^2^ Although the relative abundance of particles in the 675 to 2000 nm range is low, their scattering cross-sections are large. Truncating the long tail therefore neglects a meaningful portion of the total scattering. The relative impact of the particles exceeding 675 nm in diameter was investigated by truncating the FCM PSD at different intervals, recomputing particle number densities to remain at the target volume fraction, and evaluating the impact on the simulated optical properties.

### 2.5 Model & Experimental Comparison

To assess the proposed PYHSA model using the data inputs discussed in the previous section, we computed the optical properties for scatterer volume fractions, $\phi_{p}$, in the range 0.00227 to 0.227, corresponding to 1 to 100% v/v dilutions of stock IL20%. The concentration range was selected to coincide with the full range characterized experimentally by Aernouts et al.,^3^ including the highest volume fractions at which departures from the IS approximation are expected to be most pronounced. Optical properties were computed for the wavelength range 400 to 1700 nm. The wavelength range was selected to encompass the VIS and NIR spectral regions commonly used in biomedical optics for techniques such as tissue spectroscopy and optical coherence tomography. The simulated values were compared with the semi-empirical equations reported by Aernouts et al., which were fitted to experimental measurements for $\mu_{s}(\lambda ,\phi_{p})$ and $g(\lambda ,\phi_{p})$ in the dependent scattering regime.^3^ These empirical relations are described in Eqs. (7)–(12) below, with $\mu_{s}^{\prime }$ computed using Eq. (6). The equations below assume λ in nm and output $\mu_{s}$ and $\mu_{s}^{\prime }$ in ${\mathrm{mm}}^{-1}$. It is important to note that the empirical equations presented in Ref. 3 are only validated experimentally for the combinations of wavelength (in nm) and volume fraction satisfying the condition: $\lambda \geq 560+\phi_{p}\cdot 2200$,

$$\mu_{s}(\lambda ,\phi_{p})=\underset{\mu_{s}^{\mathrm{indep}.}}{\underset{⏟}{\left(1.868\times 10^{9}\right)\cdot \lambda^{-2.59}\cdot \frac{\phi_{p}}{0.227}}}[\frac{(1-\phi_{p}{)}^{p(\lambda )+1}}{{\left(1+\phi_{p}\cdot (p(\lambda )-1)\right)}^{p(\lambda )-1}}],$$ (7)

$$p(\lambda )=1.31+5.481\times 10^{-4}\cdot \lambda ,$$ (8)

$$g(\lambda ,\phi_{p})=\underset{g^{\mathrm{indep}.}}{\underset{⏟}{a\left(\frac{1-f}{1+e^{c(\lambda +d)}}+f\right)+b\left(\frac{1-h}{1+e^{c(\lambda +d)}}+h\right)\lambda }}+k(\lambda )\phi_{p},$$ (9)

$$[a,b,c,f,h]=[1.094,-5.653\times 10^{-4},5.3\times 10^{-3},0.3516,0.1933],$$ (10)

$$d=\frac{a(f-1)}{b(h-1)},$$ (11)

$$k(\lambda )=-10+0.05047\lambda -9.693\times 10^{-5}\lambda^{2}+8.449\times 10^{-8}\lambda^{3}\;-3.412\times 10^{-11}\lambda^{4}+5.204\times 10^{-15}\lambda^{5}.$$ (12)

Comparison with experimental data could not be performed for the phase function outside of the independent scattering regime due to a lack of available experimental data. In the independent scattering regime, the match between Mie calculations based on the PSD and refractive index data by Shahsavari et al.^12^ and experimental data was recently demonstrated by Stolz et al.^34^

### 2.6 Derivation of Descriptive Equations

To provide an accessible analytical framework for the generated optical properties, we derived, when possible, empirical equations that approximate the simulation outputs for the various optical properties. These empirical equations were obtained through nonlinear least-squares optimization. To improve numerical stability, all wavelengths were normalized to a reference $\lambda_{0}$. For the scattering phase function, such fits were not practical due to the high number of coefficients and therefore not reported. For all optical properties, we also provide the complete dataset as well as example scripts to generate interpolated values for any wavelength, concentration, and, in the case of the phase function, angle.

## 3 Results

### 3.1 PSD Binning

**Fig. 3:**
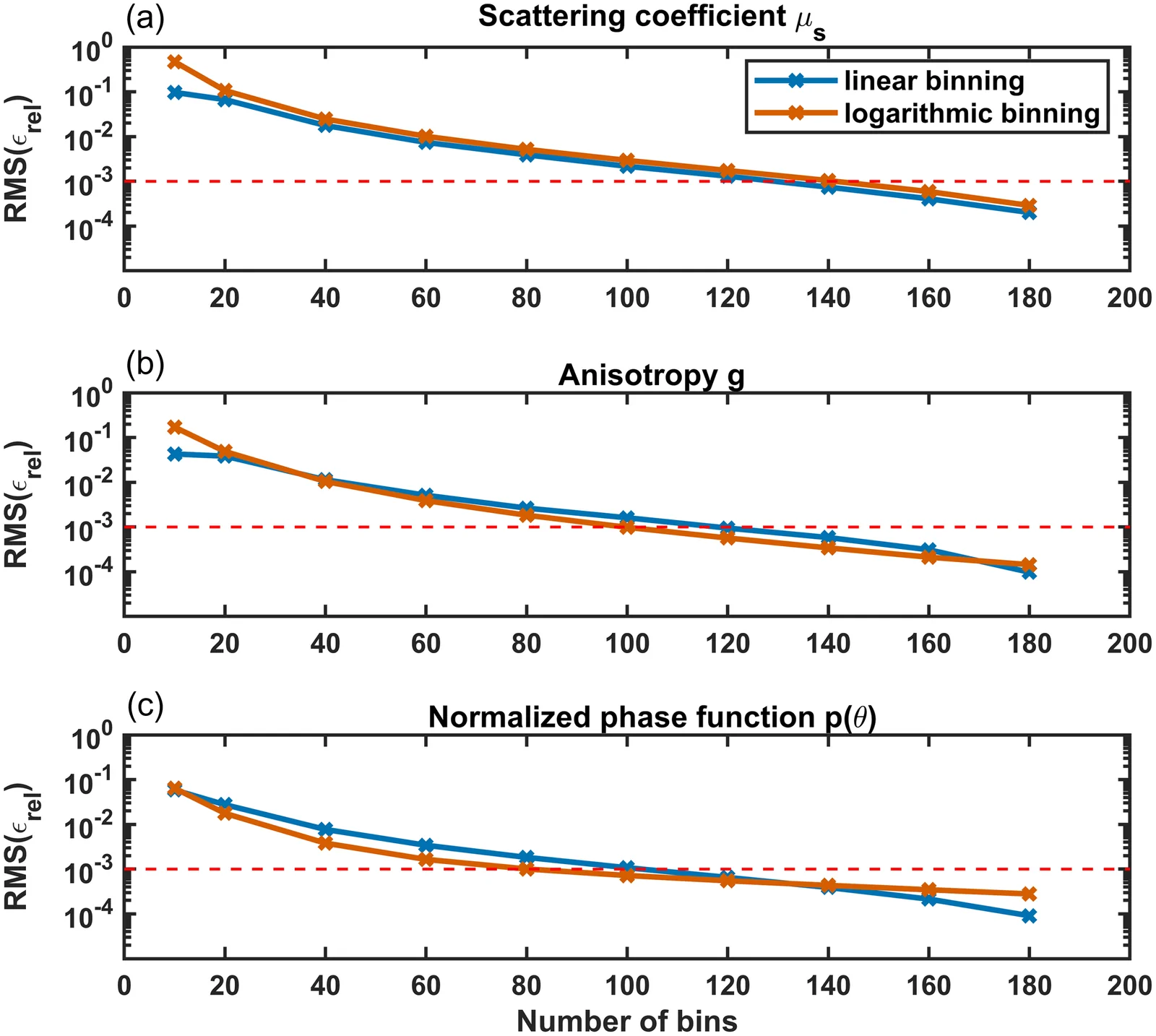
Impact of binning on computed optical properties. Root-mean-square (RMS) relative error was computed by comparing the optical property curve as a function of particle volume fraction ($\phi_{p}$) to the one computed with linear binning and $N_{\mathrm{bins}}=200$. For the phase function, RMS relative error was computed across all angles and particle volume fractions. The simulation wavelength was 600 nm. The dashed, horizontal red line in all subplots is the $10^{-3}$ or 0.1% target RMS relative error.

Figure 3 presents the impact of size binning on the computation of the optical properties of interest. The curves were generated at λ=600  nm and depict the root-mean-square (RMS) relative error when compared with the value obtained with 200 linear bins (bin width is a diameter step of ∼10  nm) across the volume fraction range $\phi_{p}\in [0.00227,0.227]$. The monotonically decreasing relative errors in all parameters indicate that the model converges to a single value. To ensure that the relative error was low ($∼10^{-3}$, indicated by the red, horizontal dashed lines), we used 140 logarithmic bins in all subsequent simulations. For reference, a 50 nm bin width equivalent to that used by van Staveren et al. corresponds to linear binning with 40 intervals, resulting in an RMS relative error of ∼1% compared with high-resolution binning.

### 3.2 PSD Truncation

**Fig. 4:**
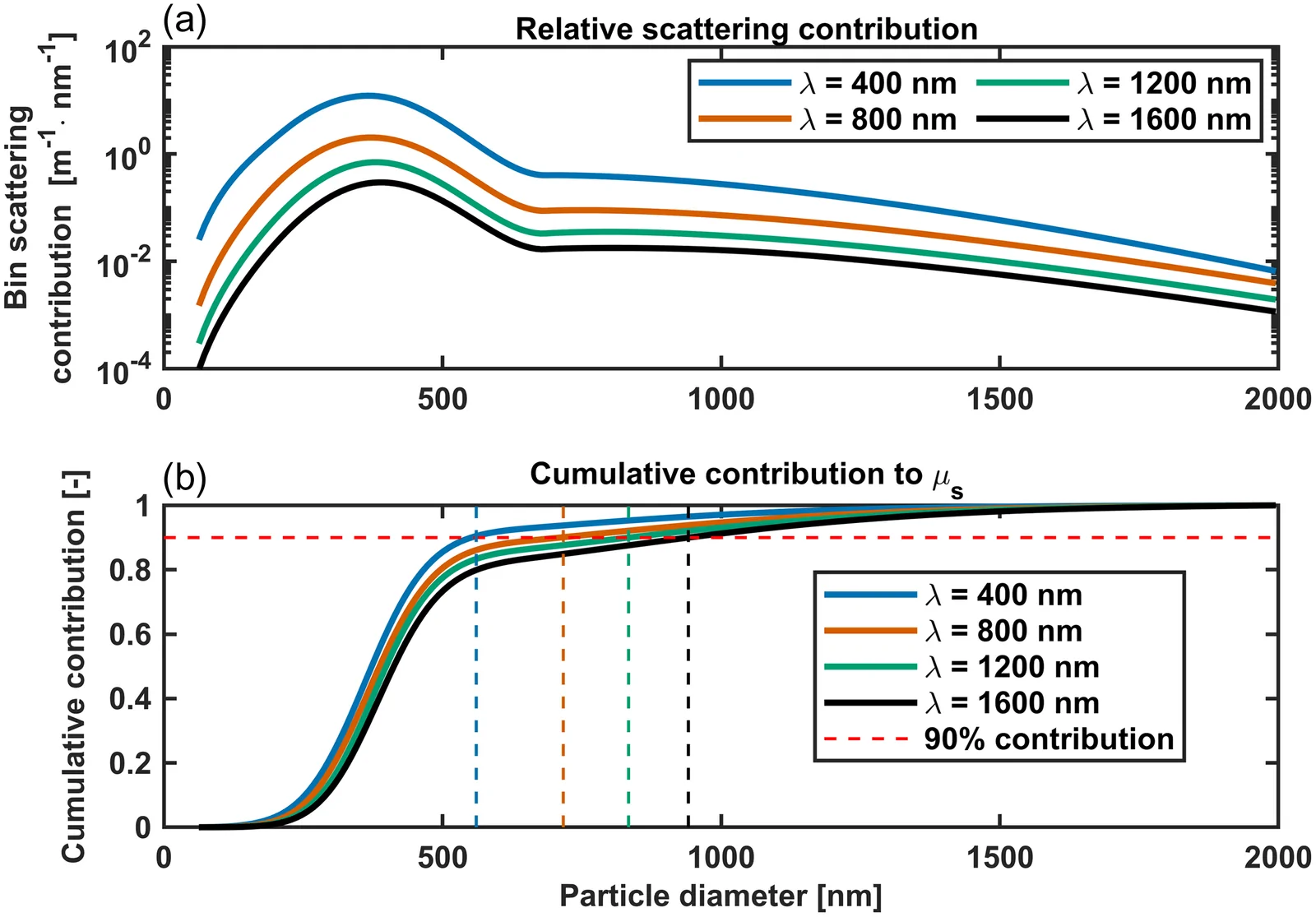
(a) Bin width-corrected scattering coefficient for each size bin. Computed as the product of the number density and the scattering cross-section obtained from Mie theory for each size bin. (b) Normalized cumulative contribution to the total scattering coefficient equal to the sum of elements of subplot (a). Vertical dashed lines indicate 90% contribution to $\mu_{s}$ for different wavelengths. Simulation performed with a volume fraction of $\phi_{p}=0.00227$ to be in the independent scattering regime and safely omit dependent scattering effects and with 140 size bins.

To highlight the importance of including the long tail in the computation of scattering properties, we first simulated a highly dilute IL solution with $\phi_{p}=0.00227$ (1% volume/volume concentration from IL20% stock). In this highly dilute solution, IS is valid, and we can compute the scattering coefficient as a simple sum of the scattering cross-sections of the different particle sizes weighted by their relative abundance [see the first part of Eq. (3)]. The result of this computation is presented in Fig. 4. Figure 4(a) shows the bin width-corrected scattering coefficient of each size bin and for different wavelengths. The main lobe of these distributions gradually shifts towards larger particle sizes as the illumination wavelength increases. This wavelength-dependent shift of the peak is explained by the fact that for increasing wavelength, smaller particles contribute less to scattering as Rayleigh scattering is proportional to $\lambda^{-4}$. As such, there is a gradual increase in the relative contribution of the larger particles for increasing wavelengths. This increasing contribution is further emphasized in Fig. 4(b), which depicts the normalized cumulative contribution to the $\mu_{s}$ values computed for each wavelength, where the dashed vertical lines indicate the particle diameter for which the cumulative contribution to $\mu_{s}$ reaches 90%. These are 560, 717, 813, and 941 nm for the wavelengths 400, 800, 1200, and 1600 nm, respectively. It is therefore apparent that the truncation at 600 to 700 nm from previously reported size distributions would result in significant underestimation of the total scattering coefficient.

**Fig. 5:**
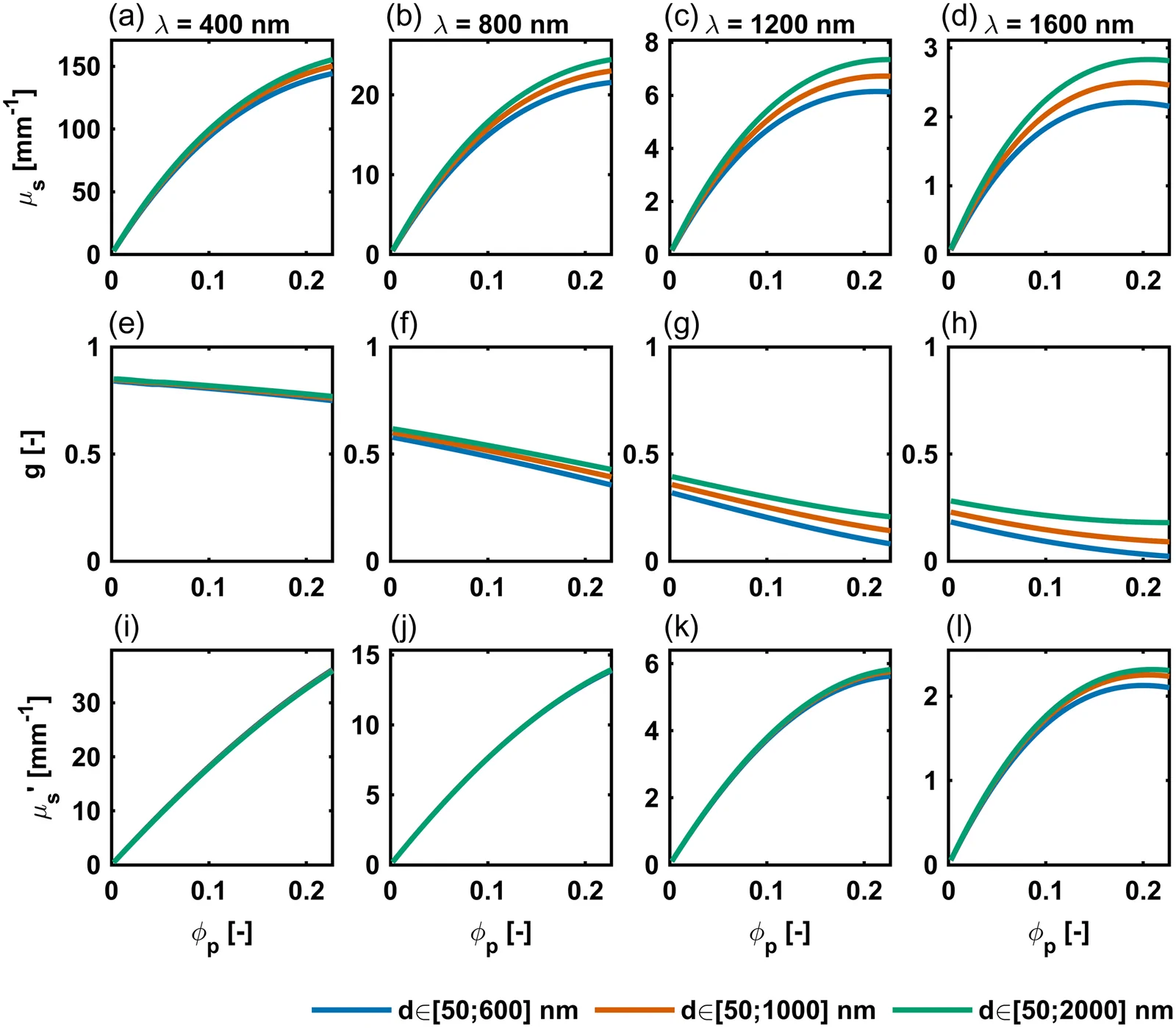
Impact of PSD truncation on the scattering coefficient, the anisotropy, and the reduced scattering coefficient, across the full $\phi_{p}$ range and for different wavelengths.

**Fig. 6:**
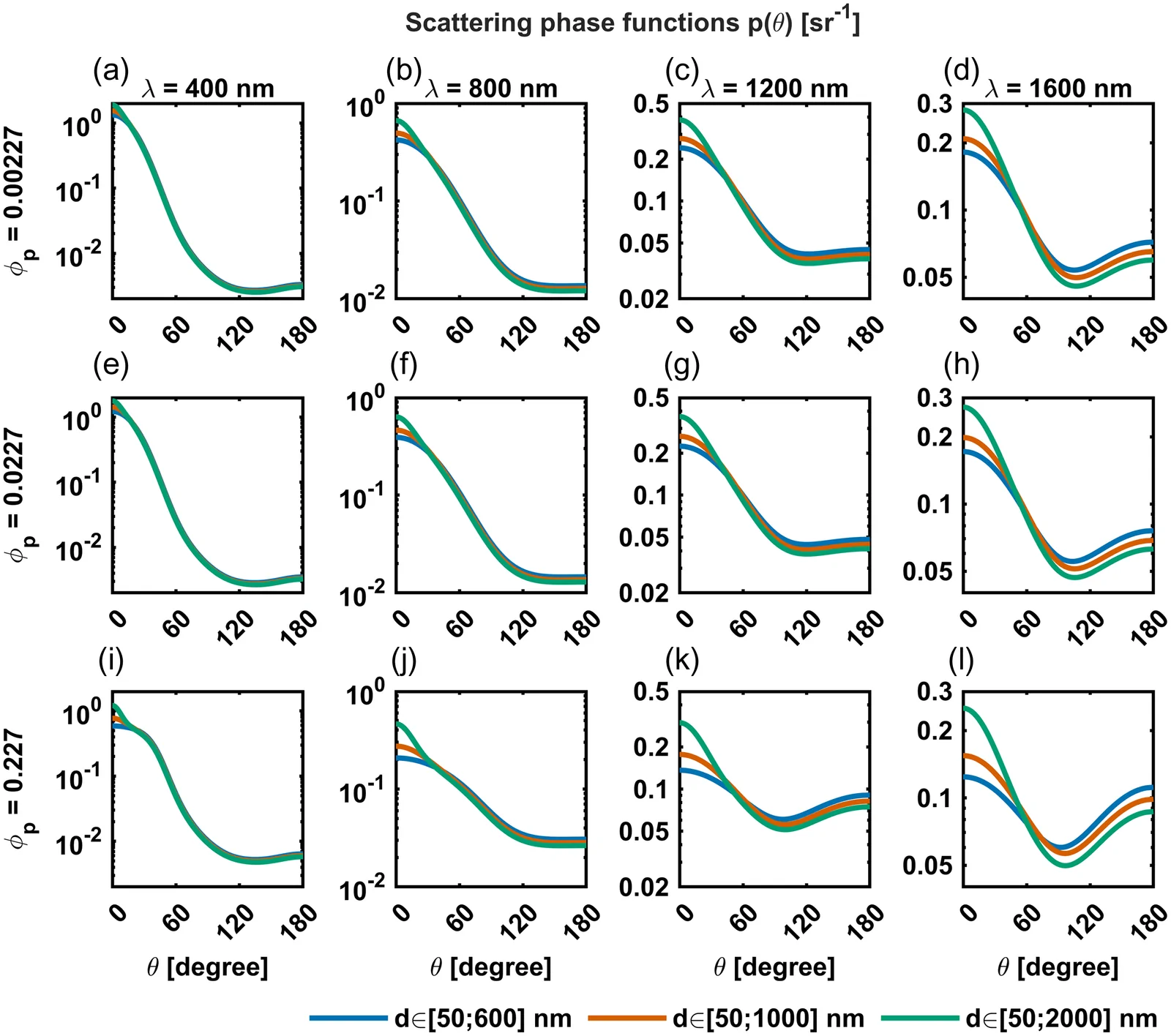
Impact of PSD truncation on the phase function. Increasing wavelength from left to right. Increasing volume fraction from top to bottom.

The truncation of the PSD also leads to effects on the scattering properties in the DS regime, as highlighted in Figs. 5 and 6, where the effects on $\mu_{s}$, g, $\mu_{s}^{\prime }$, and p(θ) are depicted. A first effect is that including the long tail strictly increases $\mu_{s}$, with the effect more pronounced for increasing wavelength. This is consistent with the observation in the IS regime depicted in Fig. 4(b). Including the long tail also strictly raises the anisotropy, which is also expected as larger particles will typically exhibit more forward-peaked scattering (Mie regime) than smaller particles (Rayleigh regime). The increased anisotropy can also be observed in the phase function, as illustrated in Fig. 6. For higher volume fractions [Figs. 6(i)–6(l)], the highly forward (θ→0  deg) and highly backward scattering (θ→180  deg) are significantly affected. For example, p (0 deg) approximately doubles at 1200 nm when the tail is included. Backscattering, p (180 deg), is also decreased by as much as 30% for high volume fraction at 1200 nm [Fig. 6(k)], and even more so for λ=1600  nm [Fig. 6(l)]. Ultimately, the observed impact of the long tail on the optical properties, particularly for NIR wavelengths, establishes its necessity for accurate modeling both under the dependent and independent scattering regimes. Interestingly, it appears that the increase in scattering coefficient and anisotropy mostly compensate for each other when considering the reduced scattering coefficient for all but the longest wavelengths, as depicted in Figs. 5(i)–5(l).

It should be noted that considering particle diameters beyond 2000 nm is not necessary as all optical properties appeared to converge to stable values. When the PSD range was extended to 3000 nm, assuming the same trend as depicted in Fig. 1, the resulting changes in optical properties were found to be negligible (data not shown).

### 3.3 Model & Experimental Comparison

**Fig. 7:**
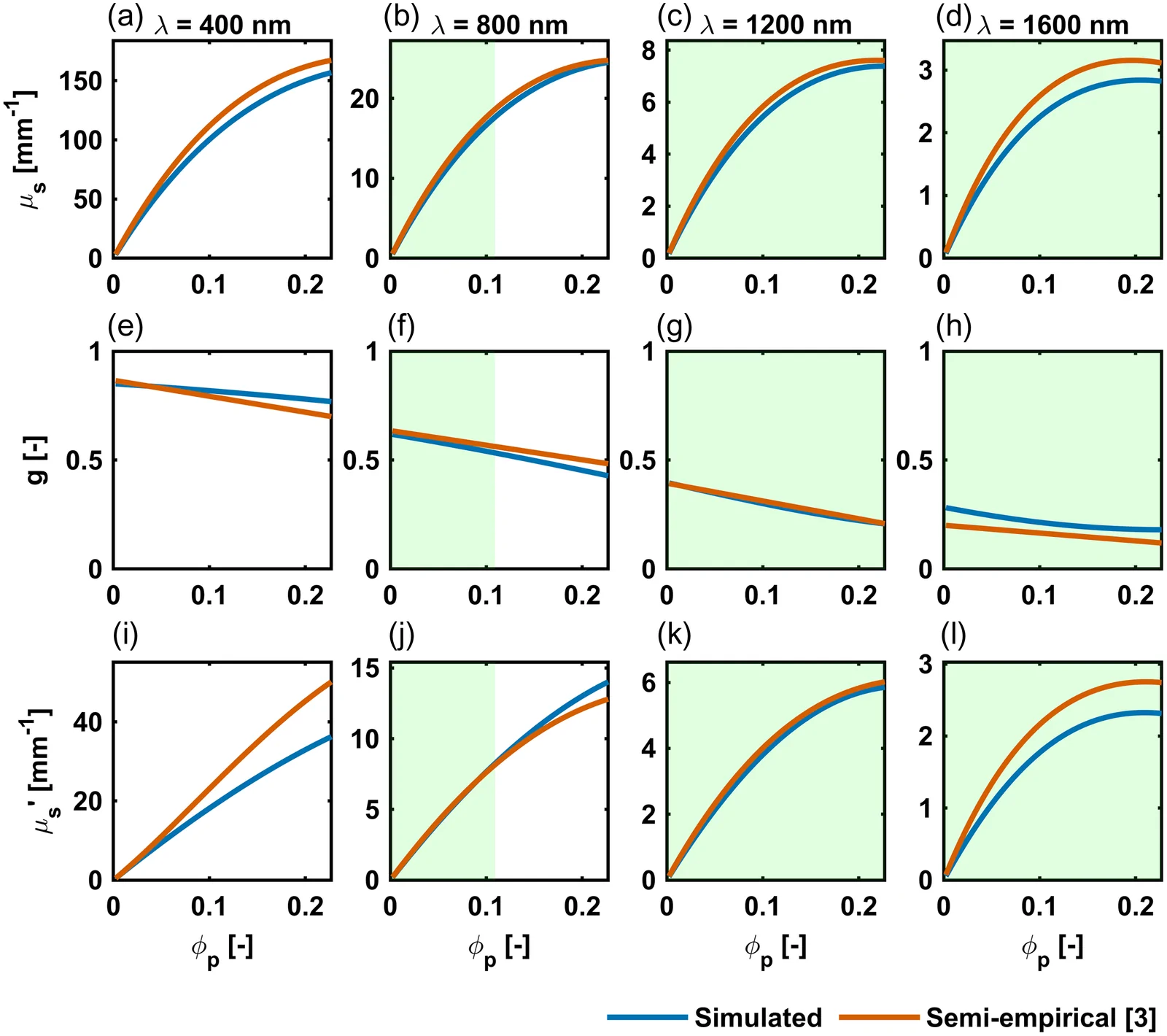
Comparison of PYHSA predictions with the semi-empirical equations fitted to experimental results by Aernouts et al.^3^ Green shaded areas indicate experimentally validated volume fraction range for the given wavelength (see Sec. 2.5).

**Fig. 8:**
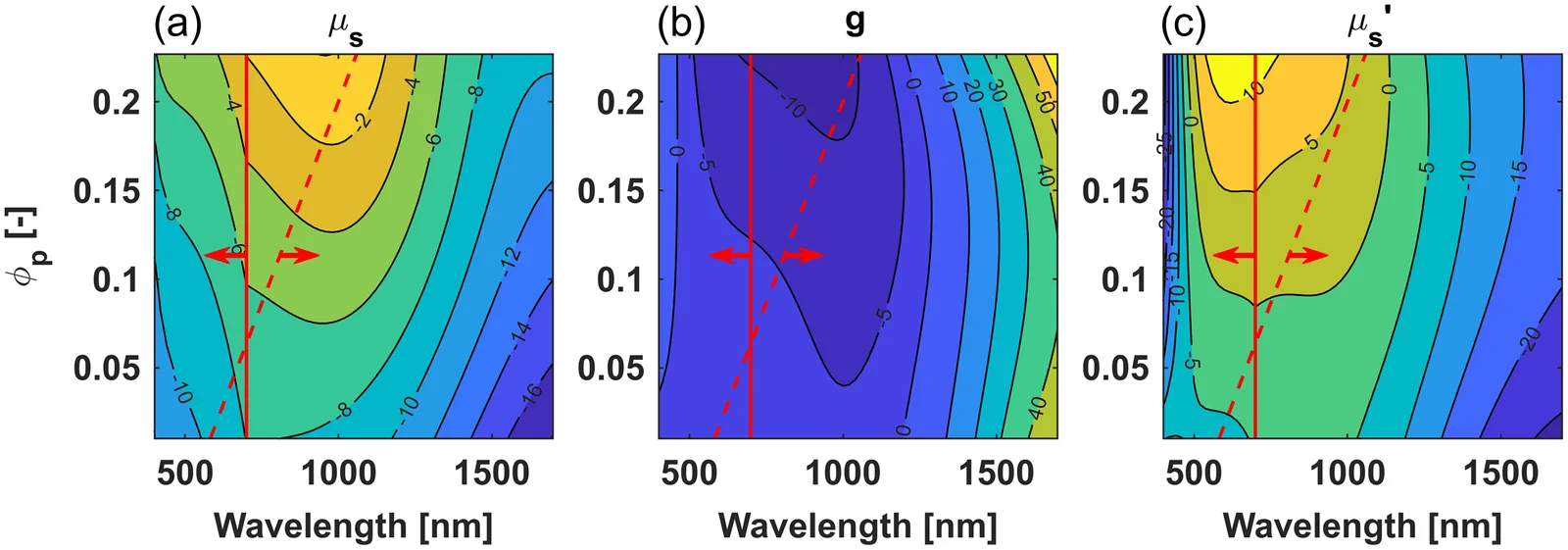
Contour plots for the relative error in % between the PYHSA model with inputs described in Sec. 2.3 and equations from Aernouts et al.^3^ The dashed red line and arrow indicate the range of validity of the semi-empirical equations. The solid red line and arrow indicate the range of validity of inputs as measured in Ref. 12.

The comparison between the optical properties computed with the PYHSA and those reported by Aernouts et al.^3^ is presented in Fig. 7 for four wavelengths and the complete range of volume fractions. Figure 7 also includes green shaded areas that indicate the range over which the equations derived by Aernouts et al. were experimentally validated. To further quantify the differences, Fig. 8 plots the relative error ($\epsilon =(X_{\mathrm{PYHSA}}-X_{\mathrm{Aernouts}})/X_{\mathrm{Aernouts}}$, where X is the chosen optical property) for all wavelengths in steps of 5 nm and volume concentrations of IL20% stock in steps of 2%. The solid red line and arrow indicate the regions of validity for high-quality refractive index data from Shahsavari et al., while the dashed line indicates the experimentally validated wavelength/volume fraction range from Aernouts et al. The RMS relative errors for $\mu_{s}$, g and $\mu_{s}^{\prime }$ are 9%, 16%, and 11% for the complete parameter space, and 9%, 2%, and 5% for the overlapping regions of validity (small triangle formed by the solid and dashed lines in Fig. 8). Overall, this indicates that the model output is broadly consistent with experimental trends, although some discrepancies remain. Although these differences are on average low (in the range of or below 10%), certain regions of the parameter space result in larger differences. For example, the anisotropy differs significantly (>20%) for wavelengths beyond 1500 nm, likely also leading to the larger differences (>15%) in reduced scattering in the same spectral range.

### 3.4 Derivation of Descriptive Equations

#### 3.4.1 Scattering coefficient

To ensure an accurate reconstruction of the simulation outputs, the independent scattering coefficient curve was modeled as a higher-order power law with respect to wavelength:

$$\mu_{s}^{\mathrm{indep}.}(\lambda ,\phi_{p})=(\frac{\phi_{p}}{0.227})\cdot a\cdot {\left(\frac{\lambda }{\lambda_{0}}\right)}^{b+c\cdot {\;\log\;}_{10}(\lambda /\lambda_{0})}.$$ (13)

The coefficients a–c were obtained by fitting $\mu_{s}(\lambda )$ in ${\mathrm{mm}}^{-1}$ computed at low concentration ($\phi_{p}=0.00227$) with a second-order polynomial in ${\;\log\;}_{10}$ space, with $\lambda_{0}=600$ nm. Fitting was performed in log-space to weight relative errors more uniformly across the spectral range, rather than allowing the larger scattering values at shorter wavelengths to dominate the optimization. This fit for the IS regime achieved an $R^{2}>0.999$ and an RMS relative error of <0.7% over the entire 400 to 1700 nm wavelength range. The dependent scattering was modeled using the same form as Eq. (7), with the updated $\mu_{s}^{\mathrm{indep}.}$. We retained the linear shape for $p(\lambda )=p_{1}+p_{2}\cdot (\lambda /\lambda_{0})$, but updated the coefficients. With these updated values, we achieved a 1.2% RMS relative error and 3.7% maximum relative error over all wavelengths and $\phi_{p}$ values. All fit coefficients are presented in Table 1.

**Table 1:** Scattering coefficient fit parameters.

a	b	c	$p_{1}$	$p_{2}$
$1.082\times 10^{2}$	−2.516	−0.4036	1.207	0.3078

#### 3.4.2 Scattering anisotropy

The scattering anisotropy in the IS regime was fitted to a (2,3) rational polynomial with respect to wavelength:

$$g^{\mathrm{indep}.}(\lambda /\lambda_{0})=\frac{n_{g,1}\cdot (\lambda /\lambda_{0}{)}^{2}+n_{g,2}\cdot (\lambda /\lambda_{0})+n_{g,3}}{(\lambda /\lambda_{0}{)}^{3}+d_{g,1}\cdot (\lambda /\lambda_{0}{)}^{2}+d_{g,2}\cdot (\lambda /\lambda_{0})+d_{g,3}},$$ (14)

achieving an RMS relative error <0.3% over the full wavelength range and $R^{2}>0.999$. The dependence of the anisotropy on the volume fraction in the DS regime was modeled using a second-order polynomial:

$$g^{\mathrm{dep}.}(\lambda /\lambda_{0},\phi_{p})=g^{\mathrm{indep}.}(\lambda /\lambda_{0})+k_{1}(\lambda /\lambda_{0})\cdot \phi_{p}^{2}+k_{2}(\lambda /\lambda_{0})\cdot \phi_{p},$$ (15)

where the curves for $k_{1}(\lambda /\lambda_{0})$ and $k_{2}(\lambda /\lambda_{0})$ are modeled as (3,4) rational polynomials expressed as:

$$k_{i}(\lambda /\lambda_{0})=\frac{n_{i,1}\cdot (\lambda /\lambda_{0}{)}^{3}+n_{i,2}\cdot (\lambda /\lambda_{0}{)}^{2}+n_{i,3}\cdot (\lambda /\lambda_{0})+n_{i,4}}{(\lambda /\lambda_{0}{)}^{4}+d_{i,1}\cdot (\lambda /\lambda_{0}{)}^{3}+d_{i,2}\cdot (\lambda /\lambda_{0}{)}^{2}+d_{i,3}\cdot (\lambda /\lambda_{0})+d_{i,4}},$$ (16)

achieving a 1.0% RMS relative error and 2.7% maximum relative error over all wavelengths and $\phi_{p}$ values. All fit coefficients are presented in Table 2.

**Table 2:** Anisotropy fit parameters.

$n_{g,1}$	$n_{g,2}$	$n_{g,3}$	$d_{g,1}$	$d_{g,2}$	$d_{g,3}$		
0.8968	−2.199	2.634	−1.460	0.2051	2.052		
$n_{1,1}$	$n_{1,2}$	$n_{1,3}$	$n_{1,4}$	$d_{1,1}$	$d_{1,2}$	$d_{1,3}$	$d_{1,4}$
0.6754	−1.546	4.182	−5.529	8.754	31.15	−50.29	31.92
$n_{2,1}$	$n_{2,2}$	$n_{2,3}$	$n_{2,4}$	$d_{2,1}$	$d_{2,2}$	$d_{2,3}$	$d_{2,4}$
−0.6857	1.129	−0.3011	−0.2391	−5.352	11.93	−11.86	4.474

#### 3.4.3 Scattering phase function

The scattering phase function obtained from the PYHSA simulations is defined over three dimensions: angle, θ, wavelength, λ, and volume fraction, $\phi_{p}$. This 3D dataset is not well described by simple, rational, or Legendre polynomials without utilizing hundreds of coefficients. Standard phase functions (e.g., simple, modified, two-term Henyey–Greenstein, and Reynolds–McCormick) also do not accurately capture the phase function shape over all wavelength and volume fraction combinations. When fitted to our simulated p(θ) curves, the best-performing phase function was the Reynolds–McCormick phase function, achieving an RMS relative error of 16% and a maximum RMS relative error of 30% over all $\left(\lambda ,\phi_{p}\right)$ combinations. More details on the performance of these conventional phase functions are provided in Appendix C.

As such, rather than reporting complex or inaccurate fits, we provide the complete dataset and scripts for 3D interpolation in the repository (https://doi.org/10.5281/zenodo.20207810). Due to the dense sampling in the dataset (5 nm steps in wavelength, 2% steps in volume/volume concentration, and 5000 angular points) and the smoothness of the curves, interpolation allows accurate extraction of the phase function values for any $\left(\theta ,\lambda ,\phi_{p}\right)$ triplet within the ranges θ∈[0,π], λ∈[400,1700] nm, and $\phi_{p}\in [0.00227,0.227]$.

## 4 Discussion

The results of this study show that combining the multicomponent PYHSA with updated particle-size and refractive-index inputs provides a physically grounded description of the scattering properties of Intralipid across the dependent-scattering regime. Three findings are central. First, the low-abundance long tail of the PSD, extending beyond the cutoff of earlier EM-based datasets, contributes nonnegligibly to the computed scattering coefficient, anisotropy, and phase function, particularly at longer wavelengths. Second, the resulting PYHSA predictions for the scattering coefficient, anisotropy, and reduced scattering coefficient are broadly consistent with the semi-empirical relations of Aernouts et al., although residual wavelength- and concentration-dependent discrepancies remain. Third, the model provides access to the full dependent-scattering phase function, for which direct experimental reference data are currently unavailable, thereby supplying a practical input for radiative-transfer and Monte Carlo simulations.

### 4.1 Interpretation of the Results

Section 3.2 highlights the relevance of the long tail in the PSD of IL. Figures 1 and 4 illustrate that excluding it leads to errors in the calculated scattering properties. Specifically, both $\mu_{s}$ and g are systematically underestimated if the long tail is not considered. Moreover, the long tail has a large impact on the overall shape of the phase function, particularly for highly forward and backward scattering angles and for longer wavelengths, where the contribution of large particles becomes more significant. Overall, these changes may have a comparatively limited impact on measurements performed in the diffuse regime (e.g., with techniques such as diffuse reflectance spectroscopy), because the changes in $\mu_{s}$ and g appear to mostly compensate each other in the reduced scattering coefficient [see Figs. 5(i)–5(l)]. More generally, these results demonstrate that number abundance alone is not a reliable indicator of optical importance in a polydisperse suspension. Although particles in the long tail represent only a small fraction of the total number of particles (∼0.1% for diameter >600  nm), their comparatively large scattering cross-sections allow them to contribute disproportionately to the bulk scattering properties. The same consideration may apply to other polydisperse particle suspensions, for which low-abundance large particles may be underrepresented because of limited counting statistics, measurement range, or PSD truncation despite having a substantial optical contribution. Their importance should therefore be evaluated based on their scattering contribution rather than their number fraction alone.

In Sec. 3.3, the outputs of our simulations are evaluated against semi-empirical relations reported by Aernouts et al.^3^ It is important to note that the comparisons in Figs. 7 and 8 are between the model output and fitted equations, and not a direct comparison to experimental data. While Aernouts et al. reported very high agreement between the fitted curves and the experimental data ($R^{2}\geq 0.992$) for $\mu_{s}$, g and $\mu_{s}^{\prime }$, these fits were performed on a limited number of data points with respect to volume fraction. Some differences between the equations and the experimental data may therefore still exist. Moreover, Eqs. (7)–(12) do not reflect the uncertainty in the experimental measurements from which they were derived, which may contribute to the residual mismatch observed. This caveat is particularly relevant for the anisotropy factor, because Aernouts et al. did not measure g independently. Rather, it was derived by separating $\mu_{s}^{\prime }$ into $\mu_{s}$ and g, using double integrating-sphere measurements in combination with unscattered-transmittance measurements. Aernouts et al. themselves note that this separation becomes less reliable when the scattering depth is too high because scattered light contaminated the unscattered-transmittance measurement or when signal levels are low due to high water absorption. These sources of uncertainty leave open the possibility that some systematic experimental error remains in the semi-empirical relations, particularly for g, as reflected in the reduced $R^{2}$ values for the linear fits of g versus volume fraction outside the 800 to 1400 nm range. Finally, it is plausible that some minor differences exist between different IL batches (e.g., based on manufacturer or production year) as suggested by variations in reported properties in different references such as Refs. 3, 7, 34, 35. As such, the agreement shown here should be interpreted as a consistency check against a widely used semi-empirical parameterization rather than an absolute validation of the PYHSA model. A rigorous validation would require direct comparison to experimental datasets including PSD, refractive index curves, documented measurement uncertainties and their propagation into confidence intervals for the fitted relationships, which would require experiments beyond the scope of the present work. For a general validation of the PYHSA beyond the use case of IL, it is likely that simpler, more controlled models such as bead suspensions would be stronger candidates.

Although the comparison in Sec. 3.3 provides a consistency check for $\mu_{s},\mu_{s}^{\prime }$ and g, it cannot assess the scattering phase function for concentrated IL, for which no experimental data are currently available. Because of this gap, simulations or model-based analyses requiring angular scattering information generally rely on the value of g together with an assumed analytical phase-function shape (e.g., Henyey-Greenstein), which may deviate from the actual phase function. The principal added value of this work is therefore the computation of a wavelength- and concentration-dependent phase function that is internally consistent with $\mu_{s},\mu_{s}^{\prime }$ and g. This added information is expected to be of limited importance for techniques operating in the diffuse regime where light transport is governed by $\mu_{s}^{\prime }$. It may, however, be of value to ballistic or sub-diffuse methods such as optical coherence tomography (OCT) and single-fiber reflectance spectroscopy (SFR) that are sensitive to specific angular regions of the phase function (e.g., angular backscattering coefficient within the detection numerical aperture, $\mu_{b,\mathrm{NA}}$ for OCT^36^ or the phase function parameter $p_{sb}$ in SFR^37^^,^^38^). Replacing an assumed analytical phase function or one derived from IS with our computed curves may reduce phase-function-induced mismatch in the forward models of these techniques, which could lead to more accurate calibration relationships and model-based recovery of optical properties.

However, the quantitative benefit of this additional information remains to be established as the phase functions in the DS regime reported in this paper remain without experimental validation. Nonetheless, several observations suggest the validity of our approach. First, a close match with experimental data (all scattering properties including p(θ)) in the IS regime as reported by Refs. 12, 34 supports the validity of the underlying PSD and dispersion properties. Second, the relative agreement of $\mu_{s}$ and g with those reported by Aernouts et al.^3^ in the DS regime suggests that the PYHSA correctly captures dependent scattering effects. As such, the phase functions derived with the PYHSA are likely to be at least approximately correct, thereby providing the community with previously inaccessible information.

### 4.2 Model & Input Parameter Limitations

The present model relies on a set of physical approximations that define its domain of validity. At the structural level, the suspension is represented as a polydisperse fluid of hard spheres, such that inter-particle correlations arise exclusively from excluded-volume interactions. This approximation neglects additional short-range forces associated with the charged phospholipid interface of the droplets. For colloidal systems, such electrostatic interactions are commonly described within the Derjaguin-Landau-Verwey-Overbeek (DLVO) framework, in which the pair potential is expressed as the sum of a van der Waals attraction and a screened electrostatic repulsion.^39^^,^^40^ In principle, these effects could be incorporated by replacing the present hard-sphere interaction model with a more general DLVO-type potential or with a hard-sphere plus Yukawa-type repulsive interaction and solving the Ornstein–Zernike equation using a closure appropriate for such soft interactions (e.g., the mean spherical approximation, hypernetted-chain closure, or Rogers–Young closure). Such extensions would account for electrostatic interactions but require additional inputs such as the surface potential and ionic strength, as well as how these quantities vary with particle size and dilution. The present hard-sphere approximation therefore remains a pragmatic simplification.

Moreover, several considerations support the hard-sphere approximation as a reasonable first-order description. As mentioned in Sec. 2 (Theory), the characteristic range of electrostatic screening in aqueous electrolytes is the Debye length, which is small compared with the particle diameters considered here. Accordingly, the main role of droplet charge is expected to be stabilization against aggregation rather than a major reorganization of the suspension microstructure. To estimate the potential influence of this neglected physics, we performed a simulation in which the electrostatic repulsion was modeled as an increase in the particle diameter for computation of the structural factors $H_{\alpha \beta }$, while keeping the optical diameters for Mie calculations fixed. Effectively, we increased the range of the steric exclusions without altering the optical responses of individual particles. This analysis, reported in Appendix A, shows that the resulting changes in $\mu_{s}$ remain minor for all wavelengths, concentrations, and tested increases in particle diameters. This further supports the approximation that electrostatic repulsion acts as a second-order perturbation to the predicted bulk scattering properties. However, this contribution appears to increase with volume fraction and will likely become more significant when considering stock solutions with higher base volume fractions, such as IL30%.

Even though the input datasets used here are of substantially higher quality than those employed in previous dependent-scattering studies, they should not be regarded as uncertainty-free. In particular, the uncertainty of the PSD along the diameter axis remains difficult to quantify rigorously. For example, the absolute measured and fitted PSDs reported in Ref. 12 yielded total particle volume fractions of 0.223 and 0.218, respectively, both below the nominal value of 0.227 for stock IL20%. This discrepancy could reflect a systematic bias in the particle sizing, but it may also indicate batch-to-batch variability, aging effects, or differences in formulation. The latter interpretation is supported by the observation in Ref. 12 that out-of-date IL bags exhibited substantial changes in reconstructed total volume fraction. Uncertainty also remains in the refractive index input: while the soybean oil measurements over 400 to 700 nm are highly precise, it is not known whether the oil measured is identical to the oil phase used in commercial Intralipid, as this information is proprietary. In addition, the optical model assumes homogeneous spherical scatterers and neglects possible deviations such as mild nonsphericity or more detailed core-shell structure, both of which are only approximately justified, especially for larger scatterers. Finally, the electromagnetic treatment also remains approximate, because the first-order electromagnetic treatment underlying the PYHSA neglects recurrent scattering, near-field coupling, and other higher-order corrections. Although these effects are expected to be limited in IL because of the weak refractive index contrast, they may contribute to part of the residual mismatch at the highest volume fractions and for the largest particles.^41^ Considering the greater contribution of larger particles for longer wavelengths as observed in Fig. 4, any residual uncertainty associated with particle geometry, tail statistics, or higher-order electromagnetic effects is also expected to become more visible in this spectral range. Overall, the combined effect of these sources of uncertainty does not invalidate the present model, but it does limit the extent to which the predicted optical properties can be interpreted as a unique, universal ground-truth description of Intralipid. Rather, the results should be viewed as a physics-based reference estimate conditioned on the selected input datasets and modeling assumptions.

### 4.3 Recommended use and Domain of Validity

The proposed model is defined over λ∈[400,1700]  nm and $\phi_{p}\in [0.00227,0.227]$, but the confidence associated with its predictions is not uniform across this entire parameter space. For the scalar scattering properties, the strongest empirical support is available within the experimentally validated domain of the semi-empirical relationships reported by Aernouts et al., $\lambda [\mathrm{nm}]\geq 560+2200\phi_{p}$. Predictions outside this domain remain physics-based model outputs, but are not directly supported by the available experimental comparison. Additional caution is warranted at the highest particle volume fractions, where the hard-sphere structural model and first-order electromagnetic treatment are most strongly tested, and at wavelengths above ∼1500  nm. At longer wavelengths, the predicted properties become increasingly sensitive to the low-abundance large-particle tail of the PSD, particle geometry, and the refractive-index inputs used in the NIR. The value λ=1500  nm should therefore not be interpreted as a strict validity cutoff but rather as the onset of a region in which the predictions are more strongly conditioned on input uncertainty and model assumptions (see Sec. 4.2 and Appendix A). This interpretation is also consistent with the larger discrepancies observed beyond 1500 nm in the comparison with the semi-empirical equations shown in Figs. 7 and 8.

## 5 Conclusion

In this work, we presented an updated physics-based model for the optical properties of Intralipid based on the multicomponent Percus–Yevick hard-sphere approximation combined with recent high-quality measurements of the particle size distribution and refractive-index dispersion. Within the scope of the adopted assumptions, the model provides a robust and practically useful description of Intralipid across a broad wavelength range and volume-fraction range and reproduces the established experimental trends in the scattering coefficient and anisotropy with generally good agreement. We provide empirical equations and lookup tables for all parameters across the entire studied parameter space.

Several directions for future work follow naturally from the present limitations. On the structural side, more elaborate interaction models could be explored to account explicitly for short-range repulsive forces associated with the charged phospholipid interface, for example, through DLVO- or Yukawa-type potentials. On the electromagnetic side, higher-order treatments that include recurrent scattering, near-field coupling, or other beyond-first-order corrections may help explain part of the residual mismatch observed at the highest volume fractions and for the largest droplets. Finally, further progress would benefit from direct experimental validation using fully characterized reference suspensions or future Intralipid datasets with documented uncertainties on the PSD, refractive index, and macroscopic optical-property measurements.

## 6 Appendix A: Sensitivity to Effective Short-range Repulsion

**Fig. 9:**
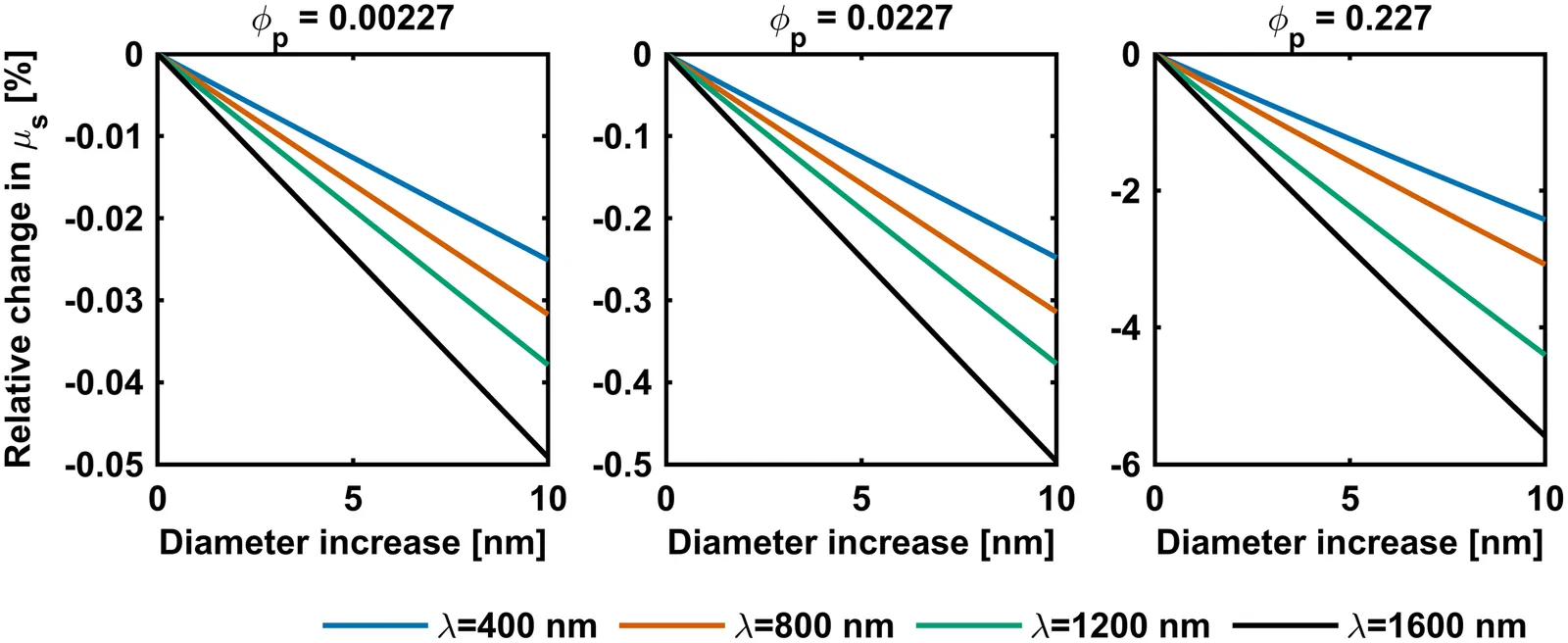
Sensitivity of the predicted scattering coefficient to an effective short-range repulsive shell added to the structural correlation model. The plotted quantity is the relative change in $\mu_{s}$ with respect to the hard-sphere baseline (0 nm increase in diameter).

As discussed in Sec. 4.2, we performed a simulation in which the particle diameter was increased for the computation of dependent scattering effects. The *optical* diameter for computing the Mie response was not altered. As illustrated in Fig. 9, we tested diameter increases up to 10 nm, corresponding to a radius increase of approximately one Debye length, as calculated in Sec. 2 (Theory). Similar variations were observed for the scattering anisotropy and scattering phase function (data not shown). Accordingly, the hard-sphere Percus–Yevick approximation appears adequate as a first-order structural model for Intralipid, while more elaborate interaction potentials should be viewed primarily as refinements for future work rather than as corrections expected to alter the main conclusions of this study.

## 7 Appendix B: Derivations

### 7.1 Derivation of $H_{\alpha \beta }(\theta )$

In this section, we report the exact equations and methods used to compute the $H_{\alpha \beta }(q)$ parameters used in Section 2.2, where q=2k sin (θ/2) is the scattering vector as a function of the wavenumber k and scattering angle θ (extracted from Ref. 20). Detailed descriptions of the underlying theory can be found in Ref. 20, in Sec. 2 of Chapter 8. All equations are implemented in MATLAB scripts in efficient vectorized form and available in the repository (https://doi.org/10.5281/zenodo.20207810) online. First, for a given size pair (i,j), the FT of the direct correlation, $C_{ij}(q)$, can be computed following:

$$C_{ij}(q)=-\frac{\pi }{6}\frac{\sqrt{n_{i}n_{j}}}{1-\xi_{3}}[M_{j}[\;\cos\;X_{i}+X_{i}\;\sin\;X_{i}+\frac{3\xi_{2}D_{i}\;\cos\;X_{i}}{1-\xi_{3}}+\frac{3\xi_{1}N_{i}}{1-\xi_{3}}+\frac{9\xi_{2}^{2}N_{i}}{{\left(1-\xi_{3}\right)}^{2}}]\;+M_{i}[\;\cos\;X_{j}+X_{j}\;\sin\;X_{j}+\frac{3\xi_{2}D_{j}\;\cos\;X_{j}}{1-\xi_{3}}+\frac{3\xi_{1}N_{j}}{1-\xi_{3}}+\frac{9\xi_{2}^{2}N_{j}}{{\left(1-\xi_{3}\right)}^{2}}]\;+M_{i}M_{j}[\frac{\xi_{0}}{1-\xi_{3}}+\frac{q^{2}\xi_{2}}{4(1-\xi_{3})}+\frac{6\xi_{1}\xi_{2}}{{\left(1-\xi_{3}\right)}^{2}}+\frac{9\xi_{2}^{3}}{{\left(1-\xi_{3}\right)}^{3}}]\;+3N_{i}D_{j}\;\cos\;X_{j}+3N_{j}D_{i}\;\cos\;X_{i}+\frac{9\xi_{2}N_{i}N_{j}}{1-\xi_{3}}],$$ (17)

where $n_{k}$ is the number density of particles with diameter $D_{k}$. The variables $M_{k}$, $N_{k}$, and $X_{k}$ are defined as

$$M_{k}=3D_{k}^{3}[\frac{\;\sin\;X_{k}}{X_{k}^{3}}-\frac{\;\cos\;X_{k}}{X_{k}^{2}}],$$ (18)

$$N_{k}=D_{k}^{2}\frac{\;\sin\;X_{k}}{X_{k}},$$ (19)

$$X_{k}=\frac{qD_{k}}{2}.$$ (20)

Finally, the coefficients $\xi_{0}-\xi_{3}$ are defined as

$$\xi_{\alpha }=\frac{\pi }{6}\sum_{k=1}^{L}n_{k}{\left(D_{k}\right)}^{\alpha },$$ (21)

where the summation runs over all L particle sizes included in the simulation. Care should be taken when evaluating $M_{k}$ and $N_{k}$ for small values of $X_{k}$ to avoid numerical errors. Use $\lim_{X_{k}\to 0}M_{k}=D_{k}^{3}$ and $\lim_{X_{k}\to 0}N_{k}=D_{k}^{2}$. From the $C_{ij}(q)$ coefficients, we can build the matrix $\bar{\bar{C}}(q)$. This matrix is included in the FT of the Ornstein–Zernike relation:

$$\overset{¯}{\overset{¯}{H}}(q)=\overset{¯}{\overset{¯}{C}}(q)+\overset{¯}{\overset{¯}{C}}(q)\overset{¯}{\overset{¯}{H}}(q),$$ (22)

where $\bar{\bar{H}}(q)$ is the matrix form of the FT of the total correlation. From Eq. (22), we can factor out $\bar{\bar{H}}(q)$ and solve for it:

$$\overset{¯}{\overset{¯}{H}}(q)={\left(\overset{¯}{\overset{¯}{I}}-\overset{¯}{\overset{¯}{C}}(q)\right)}^{-1}\overset{¯}{\overset{¯}{C}}(q),$$ (23)

where $\bar{\bar{I}}$ is the identity matrix. Each element of the $\bar{\bar{H}}(q)$ matrix, when evaluated at all q values, is the $H_{\alpha \beta }(q)$ vector used in Sec. 2.2.

### 7.2 Equations for Input Parameters

#### 7.2.1 Dispersion curves

The water and soybean oil dispersion curves for the 400 to 700 nm range follow

$$n(\lambda {)}^{2}=A_{n}+B_{n}\lambda^{2}+\frac{C_{n}}{\lambda^{2}-D_{n}^{2}},$$ (24)

with λ expressed in μm. The coefficients $A_{n}$–$D_{n}$ for both materials are given in Table 3. The values for soybean oil over the 700 to 1700 nm spectral range were extracted manually from Ref. 14 and fitted using Eq. (24). For water over the 700 to 1700 nm spectral range, we used the standard Sellmeier coefficients as reported in Ref. 33 (λ also in μm):

$$n(\lambda {)}^{2}-1=\frac{0.75831\lambda^{2}}{\lambda^{2}-0.01007}+\frac{0.08495\lambda^{2}}{\lambda^{2}-8.91377}.$$ (25)

**Table 3:** Dispersion coefficients.

Substance	$A_{n}[-]$	$B_{n}$ [$\mu m^{-2}$]	$C_{n}$ [$\mu m^{2}$]	$D_{n}$ [μm]	Ref.
Water (400 to 700 nm)	1.786850	−4.736437E-2	1.857258E-3	3.000867E-1	12
Soybean oil (400 to 700 nm)	2.126150	6.849464E-3	1.661378E-2	2.359996E-3	12
Soybean oil (700 to 1700 nm)	2.130921	−7.896278E-3	1.667742E-2	7.045986E-2	14

#### 7.2.2 Particle size distribution

The PSD used in the simulations was obtained from Ref. 12. It follows the form:

$${\;\log\;}_{10}f({Ø}_{*})=\left\{ \begin{array}{ll} A_{f}{Ø}_{*}^{4}+B_{f}{Ø}_{*}^{3}+C_{f}{Ø}_{*}^{2}+D_{f}{Ø}_{*}+E_{f}, & 57<Ø\leq 700\;\mathrm{nm},\\ F_{f}\;{Ø}_{*}+G_{f}, & 700<Ø\leq 2000\;\mathrm{nm}, \end{array}\right.$$ (26)

where ${Ø}_{*}$ is a normalized diameter defined as ${Ø}_{*}=Ø/{Ø}_{0}$ with ${Ø}_{0}=1000\;\mathrm{nm}$, and coefficients $A_{f}-G_{f}$ are given in Table 4. $f({Ø}_{*})$ denotes the dimensionless particle concentration, $f({Ø}_{*})=C({Ø}_{*})/C_{0}$, where $C_{0}$ is an arbitrary concentration of $1\;{\mathrm{mL}}^{-1}{\mathrm{nm}}^{-1}$. To convert this dimensionless PSD into histograms with true particle number densities necessary for calculation of $H_{\alpha \beta }$, first define the edges, $\left[{Ø}_{i}^{\min},{Ø}_{i}^{\max}\right]$, of each size bin i. The number density in this bin can then be obtained via integration of $f({Ø}_{*})$ following:

$$n_{i}=C_{0}\int_{{Ø}_{i}^{\min}}^{{Ø}_{i}^{\max}}f(Ø/{Ø}_{0})dØ.$$ (27)

**Table 4:** PSD coefficients.

$A_{f}$	$B_{f}$	$C_{f}$	$D_{f}$	$E_{f}$	$F_{f}$	$G_{f}$
151.5	−231.2	109.9	−22.18	12.53	−2.417	9.609

Note that the number density obtained from Eq. (27) is in units of ${\mathrm{mL}}^{-1}$ and should be converted back to $m^{-3}$ before proceeding. For each size bin defined in this manner, the particles were assumed to have a diameter equal to the center value of the bin. The number densities in all bins were then scaled by a factor γ to achieve any target volume fraction. γ was determined by solving the equation below:

$$\phi_{p}^{\mathrm{target}}=\gamma \underset{i}{\sum }n_{i}\cdot \frac{\pi {\left({Ø}_{i}^{\mathrm{center}}\right)}^{3}}{6},$$ (28)

where the summation occurs over all size bins. Particle diameters, ${Ø}_{i}^{\mathrm{center}}$, must be expressed in meters when evaluating the equation above.

## 8 Appendix C: Mismatch of Conventional Phase Functions

In this section, we quantify how well four conventional analytical phase functions reproduce the phase functions predicted by the PYHSA model. Specifically, we fitted the standard,^42^ modified,^42^ and two-term^43^ HenyeyGreenstein functions (abbreviated HG, MHG, and TTHG, respectively), as well as the Reynolds–McCormick^44^ (RMC) function, as described by Eqs. (29)–(32), to all simulated phase functions. All adjustable parameters were allowed to vary freely within their admissible ranges (β∈[0,1], $g_{i}\in [-1,1]$ and α>−0.5). The anisotropy of the fitted functions, as calculated with Eq. (5), was not constrained to match exactly that of the input PYHSA phase function,

$$p_{\mathrm{HG}}(\theta ,g)=\frac{1-g^{2}}{4\;\pi {\left(1+g^{2}-2g\;\cos\;\theta \right)}^{3/2}},$$ (29)

$$p_{\mathrm{MHG}}(\theta ,g,\beta )=\beta p_{\mathrm{HG}}(\theta ,g)+(1-\beta )\frac{3}{4\pi }{\;\cos\;}^{2}\theta ,$$ (30)

$$p_{\mathrm{TTHG}}(\theta ,\beta ,g_{1},g_{2})=\beta p_{\mathrm{HG}}(\theta ,g_{1})+(1-\beta )p_{\mathrm{HG}}(\theta ,g_{2}),$$ (31)

$$p_{\mathrm{RMC}}(\theta ,\alpha ,g_{s})=\frac{\alpha g_{s}{\left(1-g_{s}^{2}\right)}^{2\alpha }}{\pi ({\left(1+g_{s}\right)}^{2\alpha }-{\left(1-g_{s}\right)}^{2\alpha })}{\left(1+g_{s}^{2}-2g_{s}\cos\;\theta \right)}^{-(\alpha +1)}.$$ (32)

To demonstrate the inadequate match between these phase functions and our data, we performed a fitting routine for each combination of wavelength and concentration over the range λ∈[400,1700]  nm and $\phi_{p}\in [0.00227,0.227]$. For each $\left(\lambda ,\phi_{p}\right)$ combination, we minimized the solid-angle-averaged square logarithmic ratio, $L_{\Omega }$, given by

$$L_{\Omega }=\frac{1}{4\;\pi }\int_{\Omega }{\left[\ln\left(\frac{p_{\mathrm{model}}(\theta )}{p_{\mathrm{PYHSA}}(\theta )}\right)\right]}^{2}d\Omega .$$ (33)

**Fig. 10:**
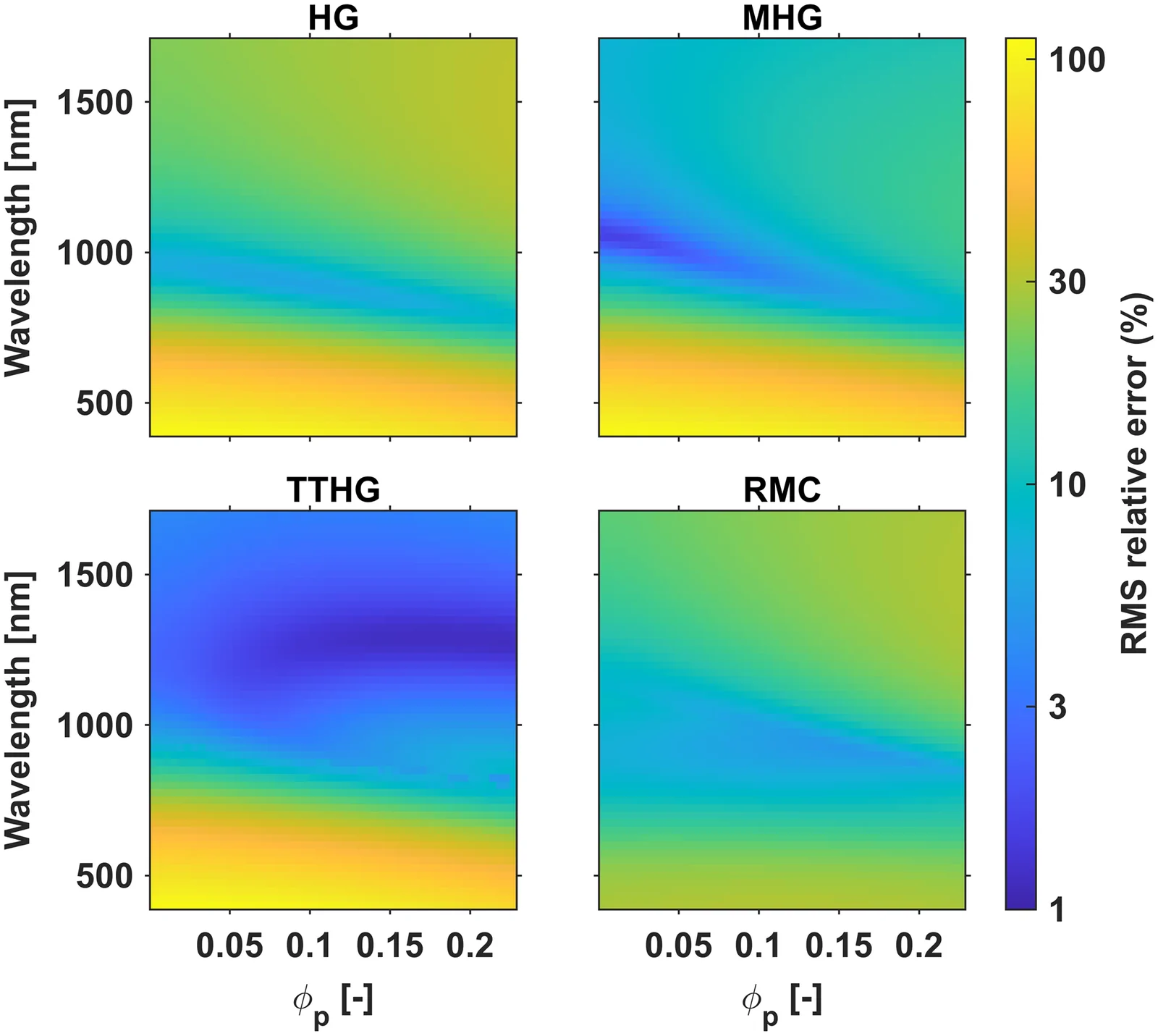
Color maps of the RMS relative error of different phase functions fitted to the simulated data produced by our method. The color scale is logarithmic, ranging from 1% to 115%.

This objective function optimizes for relative rather than absolute differences between the model and data,^45^ while the integration over solid angle ensures equal weighting per unit solid angle and avoids disproportionate contributions of the forward and backward directions. For each $\left(\lambda ,\phi_{p}\right)$ combination, we computed the RMS relative error over all angles, $\varepsilon_{\mathrm{RMS},\mathrm{rel}.}=\mathrm{RMS}[(p_{\mathrm{model}}(\theta )-p_{\mathrm{PYHSA}}(\theta ))/p_{\mathrm{PYHSA}}(\theta )]$. Figure 10 depicts this computation as color maps for the different phase functions. We found RMS relative error ranges of 6% to 110%, 2% to 110%, 1% to 110%, and 4% to 30% and global RMS relative errors [i.e., across all ($\lambda ,\phi_{p},\theta$)] of 37%, 33%, 32%, and 16% for HG, MHG, TTHG, and RMC, respectively. Moreover, we found maximum relative deviations of >500% for all HG/MHG/TTHG functions and 57% for the RMC. These ranges and Fig. 10 illustrate that the RMC is the best-performing of the four tested functions to represent our PYHSA data, but that important errors remain nonetheless. Overall, these results demonstrate that none of the tested analytical phase functions are able to reproduce the PYHSA phase functions uniformly across the complete parameter space. Finally, the large maximum deviations warrant particular caution when the fitted phase functions are used for modalities such as OCT and SFR, which are sensitive to restricted angular regions, because modest RMS relative errors do not guarantee an accurate representation of the technique-relevant portion of the phase function. As such, we recommend the use of the interpolation routines provided in the online repository (https://doi.org/10.5281/zenodo.20207810).

## Data Availability

The simulation data, lookup tables, and MATLAB scripts are publicly available in the Zenodo repository cited in Ref. 46 with the following DOI: 10.5281/zenodo.20207810.

## References

[1] Flock S. T. et al., “Optical properties of intralipid: a phantom medium for light propagation studies,” Lasers Surg. Med. 12(5), 510–519 (1992). LSMEDI 0196-8092 10.1002/lsm.1900120510 1406004

[r2] van Staveren H. J. et al., “Light scattering in intralipid-10% in the wavelength range of 400-1100 nm,” Appl. Opt. 30(31), 4507–4514 (1991). APOPAI 0003-6935 10.1364/AO.30.004507 20717241

[3] Aernouts B. et al., “Dependent scattering in intralipid^®^ phantoms in the 600-1850 nm range,” Opt. Express 22(5), 6086–6098 (2014). OPEXFF 1094-4087 10.1364/OE.22.006086 24663943

[4] Spinelli L. et al., “Determination of reference values for optical properties of liquid phantoms based on intralipid and india ink,” Biomed. Opt. Express 5, 2037–2053 (2014). BOEICL 2156-7085 10.1364/BOE.5.002037 25071947 PMC4102347

[5] Zhang X. U. et al., “Single fiber reflectance spectroscopy calibration,” J. Biomed. Opt. 22(10), 100502 (2017). JBOPFO 1083-3668 10.1117/1.JBO.22.10.100502 29086543

[6] Neubrand L. B. Attendu X. van Leeuwen T. G., “Achieving high-precision attenuation coefficient measurement in optical coherence tomography,” J. Biophotonics 18(3), e202400395 (2025). JBOIBX 1864-063X 10.1002/jbio.202400395 39821557 PMC11884974

[7] Michels René Foschum F. Kienle A., “Optical properties of fat emulsions,” Opt. Express 16(8), 5907–5925 (2008). OPEXFF 1094-4087 10.1364/OE.16.005907 18542702

[8] Di Ninni P. Martelli F. Zaccanti G., “Effect of dependent scattering on the optical properties of intralipid tissue phantoms,” Biomed. Opt. Express 2(8), 2265–2278 (2011). BOEICL 2156-7085 10.1364/BOE.2.002265 21833363 PMC3149524

[9] Aernouts B. et al., “Supercontinuum laser based optical characterization of intralipid® phantoms in the 500-2250 nm range,” Opt. Express 21(26), 32450–32467 (2013). OPEXFF 1094-4087 10.1364/OE.21.032450 24514839

[10] Fujii H. et al., “Particle size distribution effects on the light scattering properties in non-diluted colloidal suspensions: a numerical study,” Colloids Surf. A Physicochem. Eng. Asp. 703, 135208 (2024). 10.1016/j.colsurfa.2024.135208

[11] Fujii H. et al., “Photon transport model for dense polydisperse colloidal suspensions using the radiative transfer equation combined with the dependent scattering theory,” Opt. Express 28(15), 22962–22977 (2020). OPEXFF 1094-4087 10.1364/OE.398582 32752548

[12] Shahsavari M. et al., “Particle size distribution and concentration of intralipid^®^ 20%,” J. Biomed. Opt. 31(1), 015001 (2026). JBOPFO 1083-3668 10.1117/1.JBO.31.1.015001 41586154 PMC12828180

[13] Foschum F. Kienle A., “Optimized goniometer for determination of the scattering phase function of suspended particles: simulations and measurements,” J. Biomed. Opt. 18(8), 085002 (2013). JBOPFO 1083-3668 10.1117/1.JBO.18.8.085002 23974346

[14] Li X. C. et al., “Optical properties of edible oils within spectral range from 300 to 2500 nm determined by double optical pathlength transmission method,” Appl. Opt. 54(13), 3886–3893 (2015). APOPAI 0003-6935 10.1364/AO.54.003886

[15] Washington C. Koosha F. Davis S. S., “Physicochemical properties of parenteral fat emulsions containing 20% triglyceride; intralipid and ivelip,” J. Clin. Pharm. Ther. 18(2), 123–131 (1993). JCPTED 1365-2710 10.1111/j.1365-2710.1993.tb00578.x 8458880

[16] Ravagli E., “Sensor technologies for on-line monitoring of biological parameters during hemodialysis,” PhD thesis, University of Bologna (2017).

[17] Dukhin A. S. Goetz P. J., Characterization of Liquids, Nano-and Microparticulates, and Porous Bodies Using Ultrasound, Vol. 24, Elsevier (2010).

[18] Born M. Wolf E., Principles of Optics: Electromagnetic Theory of Propagation, Interference and Diffraction of Light, Elsevier (2013).

[19] Baxter R. J., “Onstein–Zernike relation and Percus–Yevick approximation for fluid mixtures,” J. Chem. Phys. 52(9), 4559–4562 (1970). 10.1063/1.1673684

[20] Tsang L. et al., Scattering of Electromagnetic Waves: Numerical Simulations, John Wiley & Sons (2004).

[21] Ding K. H. et al., “Monte Carlo simulations of pair distribution functions of dense discrete random media with multiple sizes of particles,” J. Electromagn. Waves Appl. 6(7), 1015–1030 (1992). JEWAE5 0920-5071 10.1163/156939392X00472

[22] Frenkel D. et al., “Structure factors of polydisperse systems of hard spheres: a comparison of Monte Carlo simulations and Percus–Yevick theory,” J. Chem. Phys. 84(8), 4625–4630 (1986). 10.1063/1.449987

[23] Bressel L. Hass R. Reich O., “Particle sizing in highly turbid dispersions by photon density wave spectroscopy,” J. Quant. Spectrosc. Radiat. Transfer 126, 122–129 (2013). JQSRAE 0022-4073 10.1016/j.jqsrt.2012.11.031

[24] Bressel L. Wolter J. Reich O., “Particle sizing in highly turbid dispersions by photon density wave spectroscopy: bidisperse systems,” J. Quant. Spectrosc. Radiat. Transfer 162, 213–220 (2015). JQSRAE 0022-4073 10.1016/j.jqsrt.2015.01.025

[25] Bohren C. F. Huffman D. R., Absorption and Scattering of Light by Small Particles, John Wiley & Sons (2008).

[26] Schäfer J., “Matscat,” MATLAB Central File Exchange (2024).

[27] Kodach V. M. et al., “Determination of the scattering anisotropy with optical coherence tomography,” Opt. Express 19(7), 6131–6140 (2011). OPEXFF 1094-4087 10.1364/OE.19.006131 21451637

[28] Raju M. Unni S. N., “Concentration-dependent correlated scattering properties of intralipid 20% dilutions,” Appl. Opt. 56(4), 1157–1166 (2017). APOPAI 0003-6935 10.1364/AO.56.001157 28158129

[29] van der Pol E. et al., “Standardization of extracellular vesicle measurements by flow cytometry through vesicle diameter approximation,” J. Thromb. Haemost. 16(6), 1236–1245 (2018). 10.1111/jth.14009 29575716

[30] Farkas N. Kramar J. A., “Dynamic light scattering distributions by any means,” J. Nanopart. Res. 23(5), 120 (2021). JNARFA 1388-0764 10.1007/s11051-021-05220-6 PMC11459661 39381776

[31] Driscoll D. F., “Globule-size distribution in injectable 20% lipid emulsions: compliance with USP requirements,” Am. J. Health Syst. Pharm. 64(19), 2032–2036 (2007). 10.2146/ajhp070097 17893413

[32] United States Pharmacopeial Convention, ”Globule size distribution in lipid injectable emulsions,“ in United States Pharmacopeial Convention, pp. 360–363, United States Pharmacopeia Rockville, MD (2014).

[33] Kedenburg S. et al., “Linear refractive index and absorption measurements of nonlinear optical liquids in the visible and near-infrared spectral region,” Opt. Mater. Express 2(11), 1588–1611 (2012). OMEPAX 2159-3930 10.1364/OME.2.001588

[34] Stolz L. Kienle A. Foschum F., “Characterization of temperature-dependent optical scattering of intralipid fat emulsions from 400 nm to 900 nm,” Opt. Lett. 51(7), 1943–1946 (2026). OPLEDP 0146-9592 10.1364/OL.592524 41920663

[35] Zaccanti G. Bianco S. D. Martelli F., “Measurements of optical properties of high-density media,” Appl. Opt. 42(19), 4023–4030 (2003). APOPAI 0003-6935 10.1364/AO.42.004023 12868843

[36] Almasian M. et al., “Validation of quantitative attenuation and backscattering coefficient measurements by optical coherence tomography in the concentration-dependent and multiple scattering regime,” J. Biomed. Opt. 20(12), 121314 (2015). JBOPFO 1083-3668 10.1117/1.JBO.20.12.121314 26720868

[37] van Zutphen R. van Leeuwen T. G. Attendu X., “Quantitative model for imaging single fiber reflectance spectroscopy,” Sci. Rep. 16, 17300 (2026). SRWSDA 10.1038/s41598-026-48855-y 41981093 PMC13234268

[38] Post A. L. et al., “Subdiffuse scattering model for single fiber reflectance spectroscopy,” J. Biomed. Opt. 25(1), 015001 (2020). JBOPFO 1083-3668 10.1117/1.JBO.25.1.015001 31920047 PMC7008500

[39] Derjaguin B. Landau L., “Theory of the stability of strongly charged lyophobic sols and of the adhesion of strongly charged particles in solutions of electrolytes,” Acta Physicochim. URSS 14, 633–662 (1941). ACPYAR 0365-1460 10.1016/b978-0-08-010586-4.50052-3

[40] Verwey E. J. W. Overbeek J. T. G., Theory of the Stability of Lyophobic Colloids, Elsevier (1948). 10.1021/j150453a001 20238663

[41] Mishchenko M. I. et al., “First-principles modeling of electromagnetic scattering by discrete and discretely heterogeneous random media,” Phys. Rep. 632, 1–75 (2016). PRPLCM 0370-1573 10.1016/j.physrep.2016.04.002 29657355 PMC5896873

[42] Calabro K. W. Bigio I. J., “Influence of the phase function in generalized diffuse reflectance models: review of current formalisms and novel observations,” J. Biomed. Opt. 19(7), 075005 (2014). JBOPFO 1083-3668 10.1117/1.JBO.19.7.075005 25027000 PMC4161006

[43] Pfeiffer N. Chapman G. H., “Successive order, multiple scattering of two-term Henyey Greenstein phase functions,” Opt. Express 16(18), 13637–13642 (2008). OPEXFF 1094-4087 10.1364/OE.16.013637 18772974

[44] Reynolds L. O. McCormick N. J., “Approximate two-parameter phase function for light scattering,” J. Opt. Soc. Am. 70(10), 1206–1212 (1980). JOSAAH 0030-3941 10.1364/JOSA.70.001206

[45] Tofallis C., “A better measure of relative prediction accuracy for model selection and model estimation,” J. Operat. Res. Soc. 66(8), 1352–1362 (2015). JORSDZ 10.1057/jors.2014.103

[46] de Sivry-Houle M. P. Attendu X., “MartinPdeS/PackLab: Pre-release,” (Version v0.1.0) [Computer software], Zenodo, 10.5281/zenodo.20207810 (2026).

